# AI‐Powered Mining of Highly Customized and Superior ESIPT‐Based Fluorescent Probes

**DOI:** 10.1002/advs.202405596

**Published:** 2024-07-17

**Authors:** Wenzhi Huang, Shuai Huang, Yanpeng Fang, Tianyu Zhu, Feiyi Chu, Qianhui Liu, Kunqian Yu, Fei Chen, Jie Dong, Wenbin Zeng

**Affiliations:** ^1^ Xiangya School of Pharmaceutical Sciences Central South University Changsha 410083 P. R. China; ^2^ State Key Laboratory of Drug Research Drug Discovery and Design Center Shanghai Institute of Materia Medica Chinese Academy of Sciences Shanghai 201203 P. R. China

**Keywords:** artificial intelligence, ESIPT, fluorescent probe, machine learning, virtual screening

## Abstract

Excited‐state intramolecular proton transfer (ESIPT) has attracted great attention in fluorescent sensors and luminescent materials due to its unique photobiological and photochemical features. However, the current structures are far from meeting the specific demands for ESIPT molecules in different scenarios; the try‐and‐error development method is labor‐intensive and costly. Therefore, it is imperative to devise novel approaches for the exploration of promising ESIPT fluorophores. This research proposes an artificial intelligence approach aiming at exploring ESIPT molecules efficiently. The first high‐quality ESIPT dataset and a multi‐level prediction system are constructed that realized accurate identification of ESIPT molecules from a large number of compounds under a stepwise distinguishing from conventional molecules to fluorescent molecules and then to ESIPT molecules. Furthermore, key structural features that contributed to ESIPT are revealed by using the SHapley Additive exPlanations (SHAP) method. Then three strategies are proposed to ensure the ESIPT process while keeping good safety, pharmacokinetic properties, and novel structures. With these strategies, >700 previously unreported ESIPT molecules are screened from a large pool of 570 000 compounds. The ESIPT process and biosafety of optimal molecules are successfully validated by quantitative calculation and experiment. This novel approach is expected to bring a new paradigm for exploring ideal ESIPT molecules.

## Graphical abstract

The multi‐level prediction system streamlines the ESIPT fluorophore development process by significantly narrowing the gap between design and experimentation, which translates to a substantial reduction in development cycle times.

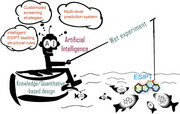

## 1 Introduction

Excited‐state intramolecular proton transfer (ESIPT) offers a unique approach for fluorescence‐based detection due to its ultrafast enol‐to‐ketone phototautomerization pathway. Fluorescent probes based on ESIPT have found widespread applications in various fields such as molecular probes,^[^ ^1^ ^]^ fluorescence sensors,^[^ ^2^ ^]^ and luminescent materials.^[^ ^3^, ^4^ ^]^ ESIPT represents a distinct class of four‐level photochemical processes, with the fluorescent moiety typically existing in the enol form (E) in the ground state. Generally, when the proton donor (‐OH, ‐NH_2_) and proton acceptor (═N‐, C═O) are in close proximity, an intramolecular hydrogen bond is formed.^[^ ^5^, ^6^ ^]^ When ESIPT molecules are excited by light, the internal charge distribution in their molecular change, consequently leading to an enhanced acidity of the proton donor and increased basicity of the proton acceptor. This triggers intramolecular proton transfer, rapidly converting the molecule from the excited state enol form (E^*^) to the excited state keto form (K^*^). Subsequently, the molecule returns to the electronic ground state through reverse proton transfer, restoring the original enol form (E).^[^ ^7^ ^]^ Compared with three well‐known mechanisms which include photoinduced electron transfer, forster resonance energy transfer, and internal charge transfer, ESIPT fluorophores exhibit strong solid‐state fluorescence and exceptional environmental sensitivity, making them valuable tools in fluorescence sensors and biological macromolecule imaging.^[^ ^8^ ^]^

However, existing research on ESIPT fluorophores mainly relies on modifying traditional fluorophores, such as HBO (2‐(2′‐hydroxyphenyl)‐benzoxazole) and HBT (2‐(2′‐hydroxyphenyl)‐benzothiazole), etc,^[^ ^9^ ^]^ which often fall short of the specific requirements for diverse applications. Moreover, the ESIPT process can be easily influenced by pressure, temperature, light, solvent polarity, pH, and ion effects,^[^ ^10^ ^]^ which increases the gap between the expectation of experiment and design. The traditional trial‐and‐error development process is not only labor‐intensive but also costly. In order to efficiently develop more promising ESIPT fluorophores, researchers have begun employing Quantitative Calculation (QC) methods to study and predict ESIPT processes. Kungwan et al. utilized the Time‐Dependent Density Functional Theory (TD‐DFT) method, selecting ‐NH_2_ type molecules with aminobenzyl or toluene benzyl as proton donors and various substituents as proton acceptors, including benzimidazole, benzoxazole, benzothiazole, or imidazopyridine, for molecular screening. They designed novel aminotype ESIPT fluorescent probes.^[^ ^11^ ^]^ Yang's group also utilized the TD‐DFT method to investigate the intramolecular excited state proton transfer process of 2‐(2′‐aminobenzyl) benzothiazole (PBTNH_2_) and three derivatives, studying the impact of introducing electron‐donating groups on the ESIPT process of aminotype hydrogen‐bonded systems.^[^ ^12^ ^]^ The classical methods of Density Functional Theory (DFT) and TD‐DFT have streamlined the design and evaluation of ESIPT molecules by eliminating the complexities of experimental procedures, providing theoretical convenience in the exploration of ESIPT mechanisms. However, the QC method is currently primarily utilized for verifying the fluorescence mechanism and is associated with substantial computational costs, but it is insufficient for discovering new fluorophores, thus we still need new approaches to address challenges in development efficiency and molecular diversity.

In recent years, with the rapid increase in biomedical data and the swift development of computer hardware and software, artifical ntelligence (AI) has profoundly influenced the advancement of the biomedical and chemical fields.^[^ ^13^, ^14^ ^]^ Particularly in the realm of drug development, AI‐based predictive models have provided innovative and precise means for chemical research, offering substantial assistance in exploring chemical reaction mechanisms, predicting molecular properties, alleviating and simplifying DFT, and conducting precise drug molecule design.^[^ ^15^, ^16^, ^17^ ^]^ Risko's team successfully utilized AI algorithms to predict the electronic, redox, and optical properties of organic π‐conjugated molecules. Compared to the several hours required for DFT, this model significantly reduced the calculation time for predicting properties to a few seconds.^[^ ^18^ ^]^ Similarly, Masato's team successfully designed large‐scale molecules with innovative fluorescence properties by combining the Molecular Generator and QC methods. Despite the need for substantial computational resources, this approach provided novel insights into fluorescence molecule design by successfully explaining the theoretical mechanisms influencing fluorescence.^[^ ^19^ ^]^ Dong et al. also employed AI algorithms to construct a multilevel model prediction framework, successfully predicting structural features and patterns of organelle‐targeting fluorescent probes. Through this method, they successfully designed and synthesized fluorescent probes with mitochondrial targeting, making a significant contribution to the development of AI in the field of fluorescent probe design.^[^ ^20^ ^]^

Based on previous research, we contemplated whether AI technology could be applied to the development of promising ESIPT molecules. This paper introduces the first utilization of AI algorithms to construct a multi‐level prediction system for developing promising ESIPT molecules. The development strategy can be divided into two parts. In the first phase, we built the first‐ever ESIPT fluorophore dataset and introduced a dual‐model system for exploring ESIPT fluorophores, which can analyze ESIPT fluorophores across a broad range from traditional small molecules to fluorophores. Candidate ESIPT molecules obtained after the prediction of the dual‐model system can be further evaluated using a regression model, which replaces DFT to predict the energy barriers required for the ESIPT process. This helps us to assess the ease or difficulty of the ESIPT process. In the second phase, we proposed three strategies to guide the development of customized ESIPT fluorophores with desirable properties of pharmacokinetics, safety and innovative structures, ensuring that the explored molecules not only undergo efficient ESIPT processes but also possess favorable safety and biodistribution profiles. Then, the multi‐level prediction system was employed to select ideal candidate ESIPT molecules from a vast pool of 570 000 candidates. The performance of our method is verified by quantitative calculation and experiment.

## 2 Results and Discussion

### 2.1 Design and Implementation of the Methodology

**Figure 1:**
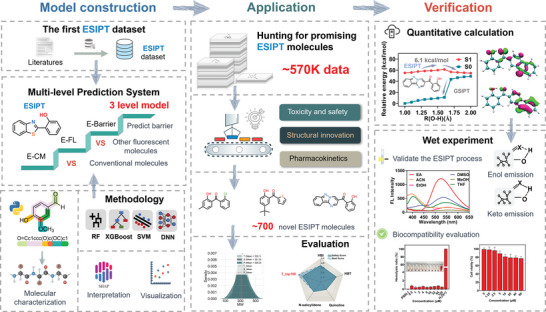
Schematic of hunting for promising ESIPT molecules with artificial intelligence, followed by quantitative calculation and wet experimental validation of the promising ESIPT molecules.

The intelligent development of promising ESIPT molecules was realized through the following stages: First, we aim to establish a multi‐level prediction model gradually increasing the discrimination difficulty. The initial level model (E‐CM) serves the purpose of filtering out compounds with potentially similar structures that are not ESIPT molecules. In the second level model (E‐CM), we employ this model to further distinguish ESIPT molecules from other fluorescent compounds, ensuring the model's focus on the specificity of ESIPT molecules. Subsequently, we utilize the third level model (E‐Barrier) model to predict the energy barrier that molecules need to overcome during the ESIPT process, thereby assessing the level of difficulty for a molecule to undergo the ESIPT process. To ensure that the explored molecules not only undergo efficient ESIPT processes but also possess favorable safety and biodistribution profiles, we have proposed a three‐strategies integrated approach to guide the development of customized ESIPT fluorophores with desirable properties: low toxicity, diverse structures, and good pharmacokinetics. For the final candidate ESIPT molecules, we further supplemented the validation of their ESIPT characteristics through QC and experimental methods, thereby establishing a tool for the accurate exploration of promising ESIPT fluorescent molecules (**Figure** **1**).

### 2.2 Dual Model Classification System: Identification of Potential ESIPT Molecules

**Figure 2:**
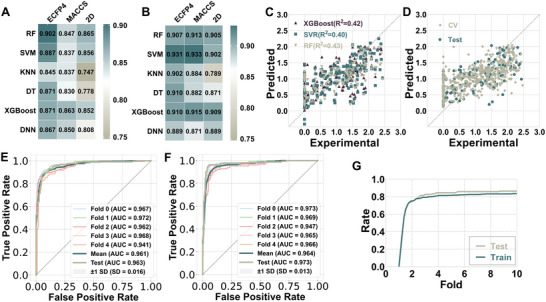
Results of models using different molecular descriptors and algorithms. The heatmap illustrates the ACC of the test set for A) E‐CM and B) E‐FL models constructed with various molecular descriptors and algorithms. C) The plot of predicted versus experimental value of the E‐Barrier model by different algorithms. D) The plot of predicted versus experimental value of the best E‐Barrier model by RF algorithm. ROC curve plots for the best E) E‐CM and F) E‐FL models in CV and the test set. G) Fold rate plot for the best E‐Barrier model.

The dual‐model classification system was developed to progressively explore potential ESIPT molecules from conventional molecules to fluorescent molecules. The first level model E‐CM model was developed to distinguish ESIPT molecules from conventional molecules. Among the different combinations of algorithms and molecular descriptors for the E‐CM model, we found that the best combination is the ECFP4‐Random Forest (RF) model with an accuracy (ACC) of 0.904 for cross‐validation (CV) and 0.902 for the test set (**Figure** **2**A and **Table** **1**). The area under the ROC curve (AUC) for CV was quite high reaching 0.961 (Figure 2E). Furthermore, 90.2% of ESIPT molecules and conventional molecules were correctly predicted in the test set (Figure S1 and Table S1, Supporting Information). The second level model E‐FL model was developed to further discriminate ESIPT molecules from other fluorescent molecules. Among the combinations of algorithms and descriptors of the E‐FL model, the best combination is ECFP4‐Support Vector Machine (SVM) (Figure 2B; Table S2, Supporting Information). This combination achieved an ACC of 0.920 for CV and 0.931 for the test set (Table 1). The AUC of 0.964 for CV showed excellent performance (Figure 2F). In addition, 97.3% of ESIPT molecules and 87.1% of other fluorescent molecules in the test set were correctly predicted (Figure S2, Supporting Information).

**Table 1:** The performance of the best E‐CM and E‐FL models.

Model	Feature	Algorithm	5‐CV					Test				
			ACC	AUC	F1	SP	SE	ACC	AUC	F1	SP	SE
E‐CM	ECFP4	RF	0.904	0.961	0.907	0.927	0.881	0.902	0.963	0.904	0.902	0.902
E‐FL	ECFP4	SVM	0.920	0.964	0.931	0.869	0.949	0.931	0.973	0.942	0.871	0.973

### 2.3 E‐Barrier Model: Accurate Assessment of the Difficulty Level for ESIPT Process

The E‐Barrier model was developed to predict the energy barriers (indicating the minimum energy required for the reaction), which can assess the difficulty level of the ESIPT process. We utilized Molecular Operating Environment software (MOE, 2018 version, Chemical Computing Group, Montreal, QC, Canada) to compute 2D and 3D descriptors for 760 molecules that had recorded energy barriers. Outlier detection was performed using ECOD from the PyOD library^[^ ^21^ ^]^ thus retaining energy barrier information for 645 molecules after excluding outliers. Following Recursive Feature Elimination (RFE) feature selection, 25 molecular descriptors were chosen to construct the E‐Barrier model. Then we employed three algorithms: RF, SVM, and eXtreme Gradient Boosting (XGBoost), along with 25 selected MOE molecular descriptors to build a series of regression models (Figure 2C). Among them, RF exhibited the best performance (Figure 2D), with an coefficient of determination (R^2^) of 0.429, achieving a 75% accuracy rate in bothCV and test set at a fold = 2 (**Table** **2**). Additionally, SVM and XGBoost also showed good results, with a 70% accuracy rate in CV at the fold = 2 (Table S3 and Figures S3–S6, Supporting Information). At present, we still have a lot of work to do to improve the performance of E‐Barrier model. In future studies, information from different solvents and 3D structures of molecules may be helpful to further improvement of the model performance.

**Table 2:** The performance of the best E‐Barrier model.

Model	Feature	Algorithm	R^2^	MAE	RMSE	Correlation	2‐fold rate	3‐fold rate
E‐Barrier	2D+3D	RF (Test)	0.384	0.463	0.568	0.626	0.749	0.821
E‐Barrier	2D+3D	RF (5‐CV)	0.429	0.449	0.548	0.660	0.753	0.795

### 2.4 Explanation Based on SHAP for Dual‐Model System

**Figure 3:**
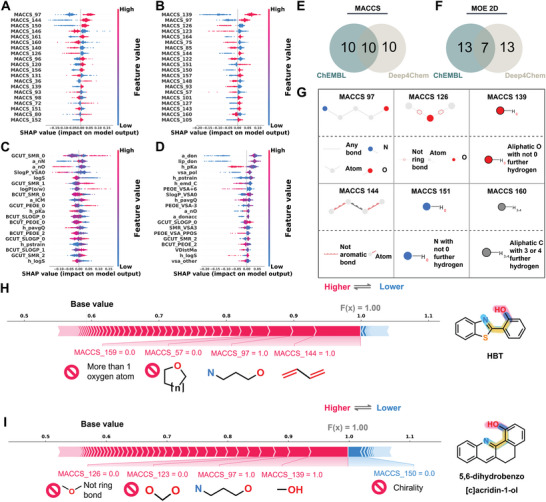
Model interpretation and structural analysis for ESIPT process. A) SHAP values for the top 20 features of the E‐CM model using MACCS‐RF. B) SHAP values for the top 20 features of the E‐FL model using MACCS‐RF. C) SHAP values for the top 20 features of the E‐CM model using 2D‐RF. D) SHAP values for the top 20 features of the E‐FL model using 2D‐RF. E) Venn diagram showing the overlapping top 20 important features in MACCS‐RF for E‐CM and E‐FL. F) Venn diagram showing the overlapping top 20 important features in 2D‐RF for E‐CM and E‐FL. G) Visualization of partially important overlapping MACCS fingerprints in E‐CM and E‐FL using MACCS‐RF. H) Conducted SHAP force diagram analysis on HBT utilizing the MACCS‐RF combination in the E‐FL model. I) Conducted SHAP force diagram analysis on 5,6‐dihydrobenzo[c]acridin‐1‐ol utilizing the MACCS‐RF combination in the E‐FL model.

To explore the relationship between molecular structure and the ESIPT process, the model explanation was performed and rules were summarized. We employed the SHapley Additive exPlanations (SHAP) method, utilizing the combination of MACCS fingerprints with the RF algorithm in the dual‐model system. For the 167 MACCS fingerprints in both the E‐CM and E‐FL models, we applied the SHAP method to separately derive the top 20 most important features (**Figure** **3**A,B). Remarkably, half of these important features were found to overlap (Figure 3E), encompassing information primarily related to hydrogen bond donors, hydrogen bond acceptors, and unsaturated bonds. The visualization of the most important MACCS fingerprints is shown in Figure 3G. In addition to MACCS fingerprints, 2D molecular descriptors have also shown excellent performance. Therefore, the 2D‐RF model was also interpreted to provide additional insights. (Figure 3C,D). From the SHAP analysis, seven descriptors were identified to overlap among the top twenty most important descriptors in both models (Figure 3F). These descriptors encapsulate information regarding charge, molecular structure, solubility, number of oxygen atoms, acidity, protonation state, and energy changes.

### 2.5 Contribution of Important Structures to the ESIPT Process

Leveraging the SHAP method, our multi‐level prediction system reveals the key substructures and molecular properties that influence the ESIPT process. We found it is very important for the ESIPT process that the molecules have proton donors and acceptors. MACCS139 and MACCS151 represent hydroxy and amino groups, respectively, serving as proficient proton donor moieties. It is noteworthy that MACCS139 holds a higher importance ranking compared to MACCS151, suggesting that hydroxy groups may be more conducive to proton donation, thereby promoting the ESIPT process. Moreover, for ‐NH_2_ type ESIPT molecules, the attachment of electron‐withdrawing groups to ‐NH_2_ is often required to enhance the proton‐donating capability, consistent with the result from the model explanation. MACCS97 and MACCS144 rank highly in both models, MACCS97 represents information about the distance between hydrogen bond donor and acceptor, and MACCS144 contains information about unsaturated bonds. Typically, the distance between proton donors and acceptors is critical for the ESIPT process. Intramolecular hydrogen bonding interactions are more facilitated when the distance between the proton donor and acceptor is closer. According to previous research on the ESIPT mechanism, in ESIPT molecules, proton donors and acceptors are usually connected through unsaturated bonds, which limits the rotation of chemical bonds to a certain extent and promotes the initiation of the ESIPT process.^[^ ^22^ ^]^ Surprisingly, model explanation using 2D descriptors revealed another critical factor influencing the occurrence of the ESIPT process: the acidity and basicity (pKa) of proton donors and acceptors. SHAP results indicated that the ESIPT process is more likely to occur when the proton donor is more acidic and the proton acceptor is more basic. However, h_pKa does not rank highly in SHAP because the calculated pKa represents the overall acidity of the entire molecule rather than the acidity of the hydrogen bond donor, thus causing h_pKa to rank lower. These findings corroborate and supplement the results obtained from previous research.^[^ ^23^ ^]^ Therefore, the acidity and basicity of the proton donor and proton acceptor as well as the distance between them should be carefully considered when designing more efficient ESIPT molecules.

To validate the practicality of the explanation and rules, we selected two classical ESIPT molecules from the training set for further analysis. Utilizing the MACCS‐RF combination in the E‐FL model, we created SHAP force diagrams for HBT and 5,6‐dihydrobenzo[c]acridin‐1‐ol, represented in Figure 3H,I, respectively. We observed that their SHAP values were both predicted as 1, indicating the high accuracy and confidence of our model in predicting classical ESIPT molecules. The segments in the force diagrams represent the contribution of different structural fragments to the model predictions, where MACCS = 1 indicates the presence of this fragment contributing to the model, and MACCS = 0 indicates the absence of this fragment contributing to the model. The structurally highlighted fragments corresponding to high contributions are annotated in the molecular structures. The results demonstrate the practicality of our explanations and rules.

### 2.6 Implementation of Three Customized Screening Strategies

**Figure 4:**
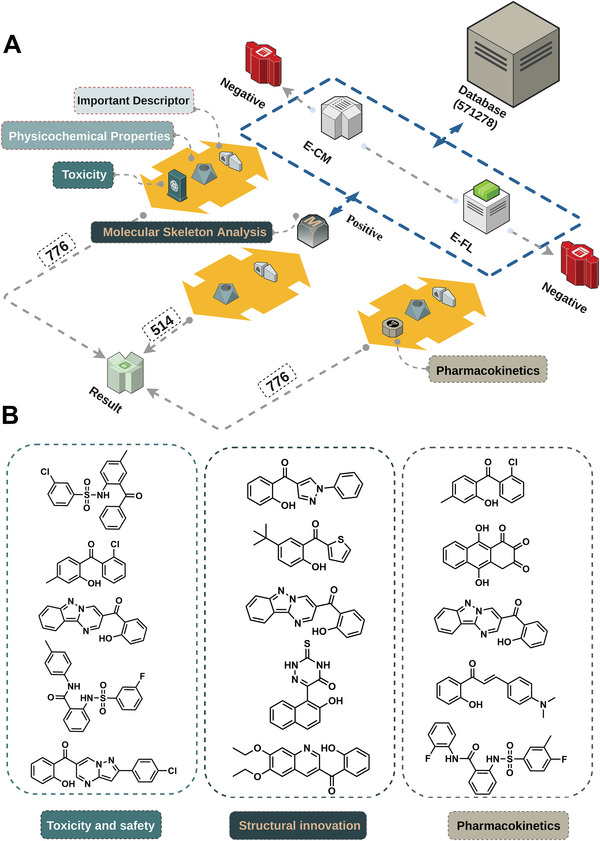
The screening of novel ESIPT molecules. A) Workflow of the multi‐level prediction system. B) Top‐ranked molecules in the screening results under different scoring strategies.

As mentioned above, in order to further verify the application value of our proposed multi‐level prediction system, we have developed three strategies to screen ideal novel ESIPT molecules from a large number of molecules (**Figure** **4**A). These three strategies are designed to ensure the ESIPT process occurs on the molecules and to meet the requirements of different applications at the same time. The scoring rules for these customized strategies include important descriptors revealed through model interpretation as well as the physicochemical properties predicted by ADMETlab 2.0.^[^ ^24^ ^]^ The undesirable toxicity is the main reason for the failure of the ESIPT‐based fluorescent probe development. Thus, we designed a safety strategy at first, the weight of safety predicted by ADMETlab 2.0 has been increased to reduce the toxic risk of the molecules, which may explore more ESIPT fluorescence sensors with lower toxicity. For most of the therapeutic agents for in vivo diagnosis and treatment, appropriate uptake into the body, reasonable distribution to various tissues and organs, metabolism without immediate loss of activity, and the establishment of suitable elimination pathways are also very important. In such cases, we designed a pharmacokinetic strategy that emphasizes the absorption, distribution, metabolism, and elimination of these molecules. In addition, although there are many ESIPT fluorescence‐based sensors with good applications in detecting ions, neutral small molecules, and biomacromolecules, the current ESIPT fluorophores still have some challenges, including ease‐of‐use, selectivity, and sensitivity, which make it difficult for current probes to meet the growing demand. This means we need to develop novel ESIPT fluorophores to expand the diversity of the existing ESIPT community. To achieve this, in the third strategy, we incorporated molecular skeleton analysis to develop novel ESIPT fluorophores with unique structures. Each strategy has corresponding customized scoring rules. The specific scoring rules and basis are shown in Tables S4–S6 (Supporting Information).

In order to implement these three customized strategies, we selected a total of 571278 molecules (molecular weight: 50–200, logP: 1–5) from the Zinc database as our molecular library for further screening. The molecular library was fed into our multi‐level prediction system to predict their ESIPT process. The prediction probability threshold was set to 0.9, and we quickly got 776 candidates from these molecules. These 776 molecules were respectively substituted into the scoring rules of the safety strategy and pharmacokinetic strategy for evaluation. For the structural innovation strategy, to capture more molecules with innovative skeletal structures, the threshold was lowered to 0.8, and 9623 molecules were obtained. Then we screened these 9623 molecules for internal molecular similarity and only retained one of the structures with a similarity greater than 0.8. Finally, we obtained 514 molecules with different skeleton structures, which were substituted into the scoring rules of the structural innovation strategy. Figure 4B shows some of the top‐ranked candidate molecules under different strategies. The top 100 results of the three evaluation strategies' scoring are provided in Tables S7–S9 (Supporting Information).

### 2.7 The Screening and Evaluation for Promising ESIPT Molecules

**Figure 5:**
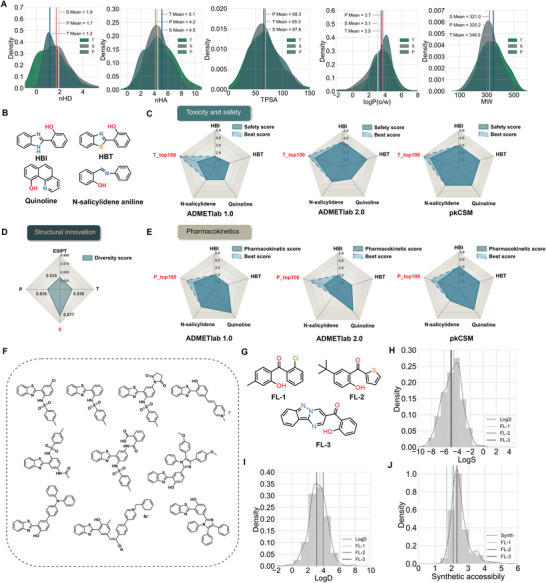
Property analysis results of candidate molecules. A) Inset (A) illustrates the top 100 essential physicochemical properties among three screening strategies. nHD represents the number of proton acceptors, nHA represents the number of proton donors, TPSA describes the physicochemical property of molecular polarity, logP indicates the partition coefficient between oil and water, and MW represents molecular weight. B) Four classic ESIPT fluorescent molecules, HBI, HBT, Quinoline and N‐salicylidene aniline. C) Comparison of the safety score of molecules ranked in the top 100 in toxicity and safety strategies with four classical fluorescent molecules. D) Comparison of the diversity score of the collected ESIPT positive set and three evaluation strategies. T: Toxicity and safety, S: Structural innovation, P: Pharmacokinetics. E) Comparison of the pharmacokinetic score of molecules ranked in the top 100 in pharmacokinetic properties strategy with four classical fluorescent molecules. F) Ten reported ESIPT molecules in our group. G) Candidate ESIPT molecules FL‐1, FL‐2, and FL‐3. H–J) Show the distribution of synthesizability, LogD, and LogS in the collected set of 922 ESIPT molecules for FL‐1, FL‐2, and FL‐3, respectively.

To screen and evaluate promising ESIPT molecules, we observed the key physicochemical properties of these potential ESIPT molecules, and we found that their relatively concentrated distribution of these properties reflects the stability of our screening strategy (**Figure** **5**A). Furthermore, to ensure the accuracy and effectiveness of our three screening strategies, we compared four classic ESIPT fluorophores (Figure 5B) with the top 100 ranked molecules using evaluation metrics and empirical decision criteria from three ADMET property prediction platforms: ADMETlab 1.0,^[^ ^25^ ^]^ ADMETlab 2.0,^[^ ^24^ ^]^ and pkCSM.^[^ ^26^ ^]^ The rating annotations are based on the recommendations of the original platform. In terms of toxicity and safety strategies, we integrated five commonly used properties, LD50, AMES toxicity, skin sensitization, hERG, and DILI, into a safety score as evaluation indicators. The safety score was incremented by +1 if these properties exhibited “Excellent,” +0.5 for “Medium,” and +0 for “Poor.” In ADMETlab 1.0, the top 100 molecules got a mean safety score of 2.5 and the best score of 5, while the other four classic ESIPT fluorophores scored in the range of 1–2. These results indicated that the safety of these top 100 molecules was better than the four classic ESIPT fluorophores. Similarly, this trend was also observed in ADMETlab 2.0 and pkCSM (Figure 5C). For the structural innovation strategy, we calculated the diversity scores of the ESIPT dataset and the molecules selected by the three strategies (Figure 5D). Apparently, the highest (0.877) diversity score of structural innovation strategies was observed compared with our training set and other strategies. Finally, in the pharmacokinetic strategy, we integrated five pharmacokinetic properties, VDss, Fu, CL, T_1/2_, and PPB as evaluation indicators. The score was incremented by +1 for “Excellent,” +0.5 for “Medium,” and +0 for “Poor”. As shown in Figure 5E, the top 100 molecules got an average pharmacokinetic score of 3 and the best score of 4 in ADMETlab 1.0, which were better than those classic fluorophores except quinoline. The structure of quinoline is quite simple, so the good VDss and Fu may result in a comparable score. Similar trends also can be found in ADMETlab 2.0 and pkCSM (Figure 5E). Overall, the comprehensive evaluation of molecules selected through these three screening strategies shows good performance on various ADMET prediction platforms.

After evaluating the molecules obtained from the three strategies, we aimed to select some preferred molecules for further validation. At this point, we should not only focus on molecules with higher scores among the three strategies but also consider some important physicochemical properties. Because the optimal ones will be synthesized for experimental validation, synthetic accessibility as an important indicator balancing results and costs should be considered. LogD and LogS are respectively associated with the distribution of molecules in lipid and aqueous phases, which determines the ease‐of‐use of molecules and their potential applications in the body or organs. Taking into account the factors mentioned above, we ultimately selected FL‐1, FL‐2, and FL‐3 as preferred molecules (Figure 5G). It is worth noting that the three molecules were not previously reported for their ESIPT properties. Figure 5H–J display the distribution of synthesizability, LogD, and LogS in the collected set of 922 ESIPT molecules for FL‐1, FL‐2, and FL‐3. It was shown that all the selected compounds exhibit good lipophilicity and hydrophilicity with acceptable synthetic accessibility.

Additionally, we tested the performance of our multi‐level prediction system in practical applications. We predicted a set of ten ESIPT molecules previously reported by our team (Figure 5F; Table S10, Supporting Information). It should be noted that these ten molecules were not included in the modeling dataset. After prediction, all the molecules were labeled as ESIPT molecules in the Dual‐Model System, which indicated a 100% recovery rate. What's more, the average scores of these ten molecules were within the top 50%, 43%, and 52% of the candidates in the three screening strategies, respectively. The average scores of the ten ESIPT molecules reported in three evaluation strategies can be found in Figure S7 (Supporting Information). This external validation not only demonstrated the accuracy of our system but also provided strong evidence for the exploration of potential ESIPT molecules in the real world.

### 2.8 Quantitative Calculations of Candidate Molecules to Validate the ESIPT Process

**Figure 6:**
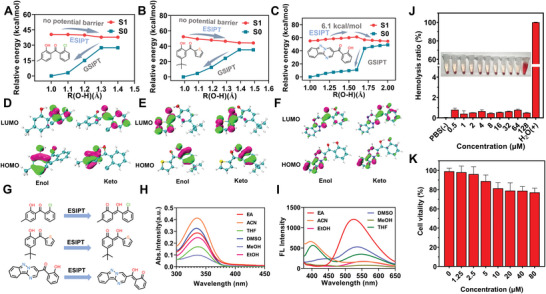
Validation results of candidate ESIPT molecules. A) S1 state energy distribution of FL‐1 along the O‐H distance in DMSO solution, optimized using PBE0/Def2‐svp. B) S1 state energy distribution of FL‐2 along the O‐H distance in DMSO solution, optimized using PBE0/Def2‐svp. C) S1 state energy distribution of FL‐3 along the O‐H distance in DMSO solution, optimized using PBE0/Def2‐svp. D) The LUMO and HOMO of molecule FL‐1 in the S1 state and the corresponding transition energies at the PBE0/Def2‐svp/IEFPCM levels. E) The LUMO and HOMO of molecule FL‐2 in the S1 state and the corresponding transition energies at the PBE0/Def2‐svp/IEFPCM levels. F) The LUMO and HOMO of molecule FL‐3 in the S1 state and the corresponding transition energies at the PBE0/Def2‐svp/IEFPCM levels. G) Schematic representation of the ESIPT process for FL‐1, FL‐2, and FL‐3. H) Absorption spectra of FL‐3 in different solvents. I) Emission spectra of FL‐3 in different solvents. J) Hemolysis percentage of red blood cells (RBCs) treated with different concentrations of FL‐3. K) Cell viability of A549 cells incubated with FL‐3.

In order to evaluate the difficulty of FL‐1, FL‐2, and FL‐3 undergoing the ESIPT process, we used the E‐Barrier model to predict their energy barriers. The predicted barriers for ESIPT processes were 1.2, 1.5, and 2.3 kcal mol^−1^ for FL‐1, FL‐2, and FL‐3, respectively. The results indicate all three molecules can undergo ESIPT process. We further scanned the potential energy curves (PEC) for the ESIPT processes of FL‐1, FL‐2, and FL‐3 in DMSO solution, as shown in **Figure** **6**A–C. Surprisingly, the potential energy curves and front molecular orbital calculations for these molecules indicated their successful ESIPT process, and the difficulty of ESIPT process is consistent with the predictions from the E‐Barrier model. In the S1 state, FL‐1 and FL‐2 show no potential barriers, while FL‐3 in the S1 excited state needs to overcome a 6.1 kcal mol^−1^ barrier for the ESIPT process. Compared with FL‐1 and FL‐2, the ESIPT process of FL‐3 will be slightly slower, which is consistent with the prediction results of the E‐Barrier model. The schematic diagram of FL‐1, FL‐2, and FL‐3 transitioning from enol to keto forms is shown in Figure 6G. The visualization of the enol and keto forms from HOMO to LUMO in the S1 state for FL‐1, FL‐2, and FL‐3 is depicted in Figure 6D–F, with the transition orbitals defined as π character to π* character, representing commonly allowed electronic transitions.

### 2.9 Synthesis of Candidate Molecules to Validate the ESIPT Process

Based on the comprehensive assessment and computational results, FL‐3 consistently ranked high in all three scoring strategies. Therefore, considering the synthesis feasibility under experimental conditions and the analysis of FL‐3′s solvent effect, we synthesized FL‐3 and validated its spectral properties (Figures S8–S10, Supporting Information). The absorption and emission spectra of FL‐3 in different solvents are shown in Figure 6H,I, with the maximum absorption peak at 335 nm. Under 335 nm excitation, emissions from both the enol and keto forms were observed (Table S11, Supporting Information), confirming that FL‐3 undergoes the ESIPT process upon photoexcitation. However, the emission ratio of enols to ketones varies in different solvents, which also shows that after exploring new ESIPT molecules, environmental factors can be rationally manipulated to control the ESIPT process.

### 2.10 Biocompatibility Evaluation of Candidate Molecules

Hemolysis and cytotoxicity are both important indicators to judge the biocompatibility of compounds. As shown in Figure 6J, no obvious hemolysis was observed in the tested concentration range. In addition, FL‐3 had no significant effect on the activity of A549 cells. Even at a concentration of 80 µM, ≈77% of A549 cells remained viable (Figure 6K). These findings are consistent with the results obtained from our methodology and demonstrate the favourable biocompatibility of FL‐3. Through these validation results, we have demonstrated FL‐3 as the optimal selection by using various theoretical calculations and experimental methods, while also confirming the effectiveness of our approach.

## 3 Conclusion

In summary, a new methodology designed for rapid and efficient exploration of promising ESIPT molecules driven by artificial intelligence was first proposed. We built the first‐ever ESIPT fluorophore dataset and established a multi‐level prediction system for exploring ESIPT molecules by systematically comparing advanced algorithms and various molecular representations. Then, key substructures and molecular properties that influence the ESIPT process were analyzed by model interpretation. Not only were the patterns consistent with published studies successfully revealed, but also interesting rules that had not been reported. For example, the conjugate double bond and the imino group also had a facilitative effect on the ESIPT process, while the presence of an oxygen‐containing heterocyclic ring had a negative effect. These findings provide important references for further study on the ESIPT mechanism and molecular structure modification. Furthermore, three screening strategies guiding the development of customized ESIPT fluorophores were implemented, ensuring good safety, pharmacokinetic properties, and novel structures. Based on our system and strategies for pharmacokinetics and toxicity, 776 molecules exhibiting unreported ESIPT phenomena were rapidly screened out from a vast pool of 570 000 candidates, respectively. Detailed analysis indicated that their safety and pharmacokinetic properties were outstanding compared with classic ESIPT fluorophores. For the third strategy, 514 ESIPT candidates with different skeleton structures were obtained. The diversity scores showed better structural diversity than the known ESIPT dataset and the other two strategies. This demonstrated that our proposed prediction system combined with these strategies can be an effective way to discover novel ESIPT molecules for different scenarios. What's more, for the selected preferred compounds, quantitative calculations and experiments validated their capability to undergo ESIPT process with good biocompatibility. In the future, there is still more work that can be done to improve our methodology. The high sensitivity of ESIPT emission to its local surroundings suggests that enlarging the data size of the prediction system can expand its application domain. In addition, the complex environmental or experimental factors are worth further investigation to clarify their relationship with ESIPT, thereby considering incorporating these factors to enhance the accuracy of the model. We believe that this research not only provides a powerful approach for the development of ESIPT molecules but also establishes a new paradigm to make a step forward in the intelligent design of molecular probes.

## 4 Experimental Section

### Data Preparation

The quality of data determines the quality of the model. From over 2700 papers in recent years, a total of 1003 ESIPT molecules were manually collected to build the dataset and support the design of the model system. To ensure the quality of the data, these molecules were subjected to a series of processing steps. First, the MOE was used to inspect all molecules and converted them into SMILES for deduplication to ensure data uniqueness. After the deduplication process, 922 ESIPT molecules were retained. Additionally, within these 922 ESIPT molecules, 760 molecules recorded the energy barriers required for ESIPT occurrences. The distribution of Proton acceptor (nHA), Proton donor (nHD), Molecular Weight (MW), LogP, and Topological Polar Surface Area (TPSA) for the collected molecules is shown in Figure S11 (Supporting Information). In the initial layer model (E‐CM), the study opted to retrieve conventional small molecules from the ChEMBL database^[^ ^27^ ^]^ as the negative set. In the second layer model (E‐FL), the choice for the negative set involved retrieving fluorescent small molecules from the Deep4Chem database.^[^ ^28^ ^]^ In ChEMBL, millions of small molecules were collected with molecular weights ranging from 100 to 600, and in Deep4Chem, all 6810 molecules available were gathered. In order to avoid significant differences in the data volume of positive and negative samples, MACCS molecular fingerprints were used to compare the similarity of the 922 manually collected ESIPT molecules with those in ChEMBL and Deep4Chem. The study set a 95% similarity threshold for molecules in ChEMBL and 80% for molecules in Deep4Chem. Finally, the study got 915 molecules in CHEMBL and 633 molecules in Deep4Chem as negative samples. For the E‐Barrier model, the study utilized 760 ESIPT molecules with barrier information from the positive samples as the training dataset for the regression model. All collected barriers were converted to units of kcal mol^−1^.

### Molecular Descriptors and Fingerprints

In this work, various types of molecular descriptors and fingerprints were employed to represent the physicochemical properties and structural features of molecules. Representative 2D and 3D molecular descriptors were provided by the MOE, comprising 206 continuous and 148 discrete descriptors. ECFP4 fingerprints and MACCS fingerprints were provided by PyBioMed.^[^ ^29^ ^]^ These molecular descriptors and fingerprints encompassed a wealth of physicochemical and topological information, including details about atoms, bonds, and rings, and additional information about these fingerprints can be found in ChemDes.^[^ ^30^ ^]^ To reduce noise and enhance the quality of the model, data preprocessing was performed on the MOE 2D and 3D descriptors by: 1) removing descriptors with zero variance, and 2) eliminating highly correlated descriptors (>0.95).

### Machine Learning Algorithms

In order to explore the most suitable machine learning model, algorithms such as K‐Nearest Neighbors (KNN),^[^ ^31^ ^]^ Decision Tree (DT),^[^ ^32^ ^]^ RF,^[^ ^33^ ^]^ SVM,^[^ ^34^ ^]^ XGBoost,^[^ ^35^ ^]^ and Deep Neural Network (DNN)^[^ ^36^ ^]^ were utilized to build models. These algorithms, varying in simplicity or complexity and grounded in different principles, possess distinct advantages and have been successfully applied in various scenarios. They were implemented in a Python (3.7.2) environment equipped with scikit‐learn (1.0.2) and XGBoost (1.6.2). Models utilizing classical algorithms were trained using a random split of 75% of the dataset for training and the remaining 25% for testing to evaluate model performance. Additionally, a five‐fold CV was applied to the training set to ensure the robustness of the models.

### Model Evaluation Metrics

For classification models, five metrics were chosen to evaluate the performance of the models: ACC, Specificity (SP), Sensitivity (SE), F1 Score (F1), and AUC.

(1) $$ACC=\frac{TP+TN}{TP+TN+FP+FN}$$

(2) $$SP=\frac{TN}{TN+FP}$$

(3) $$SE=\frac{TP}{TP+FN}$$

(4) $$F1=\frac{2\times TP}{2\times TP+FP+FN}$$

(5) $$AUC=\int_{0}^{1}\frac{TP_{i}}{TP_{i}+FN_{i}}\times \left(\frac{FP_{i}}{FP_{i}+FN_{i}}\right)d\frac{FP_{i}}{FP_{i}+FN_{i}}$$

For regression models, three metrics were employed to assess the performance of the models: R^2^, Mean Absolute Error (MAE), and Root Mean Squared Error (RMSE).

(6) $$R^{2}=1-\frac{{\sum \left({\hat{y}}_{i}-y_{i}\right)}^{2}}{\sum {\left(y_{i}-\bar{y}\right)}^{2}}$$

(7) $$MAE=\frac{1}{N}\sum_{i=1}^{N}\left|{\hat{y}}_{i}-y_{i}\right|$$

(8) $$RMSE=\sqrt{\frac{1}{N}\sum_{i=1}^{N}{\left({\hat{y}}_{i}-y_{i}\right)}^{2}}$$

Energy barriers are typically expressed in units of kcal mol^−1^. Generally, for ESIPT molecules, if the energy barrier exceeds ≈15–20 kcal mol^−1^, it may be considered that the ESIPT process cannot occur.^[^ ^37^ ^]^ This judgment is based on the results of experimental and theoretical research, but it is important to note that there may be a certain margin of error in practical situations. Therefore, predictions within a range of different‐fold errors (Folds) were utilized for compound energy barriers. It is considered to be a successful prediction when the fold < 2.^[^ ^38^, ^39^ ^]^

(9) $$\mathrm{fol}d_{i}=1+\frac{\left|{\hat{y}}_{i}-y_{i}\right|}{y_{i}}$$

A higher rate at a fold represents a better performance of the model.

Here, TP represents true positive, TN represents true negative, FP represents false positive, and FN represents false negative. N is the number of samples, $\hat{y_{i}}$ and *y_i_* are the predicted and experimental values of the i_th_ sample, and $\bar{y}$ is the mean value of the N samples.

### Feature Selection and Explanation

Preprocessing and feature selection are necessary for the calculated molecular descriptors to avoid redundancy and enhance model interpretability. Therefore, RFE^[^ ^40^ ^]^ was employed for feature selection of MOE molecular descriptors. In this study, all models utilizing MOE molecular descriptors underwent feature selection using RF‐based RFE, resulting in the identification of an optimal set of descriptors (Figures S12–S14, Supporting Information).

To interpret the predictive results of the model, the study employed SHAP, a widely used method for interpreting machine learning models.^[^ ^41^ ^]^ SHAP values were utilized to quantify the contribution of each feature to the predictions, revealing the directional impact of individual features on the outcomes. This approach aids in understanding the associative relationship between the properties of fluorescent molecules and their structures.

### Methods of Theoretical Calculation

This study conducted theoretical calculations and result analysis at the DFT and TD‐DFT levels using Gaussian 16.^[^ ^42^ ^]^ For the structures preceding the ground state, MOE was employed for conformational searches to obtain the lowest‐energy conformation, utilized for subsequent ground state optimization. The calculations were performed at the PBE0/Def2‐svp level for the optimization of the ground state (S0) and the first excited state (S1).^[^ ^43^ ^]^ The IEFPCM^[^ ^23^ ^]^ solvent model was used, with dimethyl sulfoxide (DMSO, dielectric constant (ε) is 46.826) as the solvent. The potential energy surfaces (PES) in the S0 state and S1 state were established by varying the O‐H bond distance to a series of values to estimate the ESIPT process. The computational results were executed and visualized using the Multiwfn software^[^ ^44^ ^]^ and VMD program.^[^ ^45^ ^]^

### Methods and Materials of Experiment

The benzoic acid and other reagents used in the experiment were purchased from China Energy Chemical Co., Ltd., and commercial suppliers do not need further purification. The reaction progress was monitored by thin‐layer chromatography (TLC) using 0.25 mm silica gel plates (60F‐254) and UV light (254 or 365 nm). For fluorescence and UV–vis measurements, a 10 mM stock solution of the fluorescent substance was prepared before each experiment. The fluorescence measurements were conducted with excitation at the strongest absorption wavelength, recording emission spectra in the range of 350 to 650 nm. UV–vis spectra were recorded in the range of 300–450 nm. All experiments were conducted at room temperature.

### Hemolysis Assay

Healthy mice blood (2 mL) was donated from female mice. RBCs were collected by centrifugation at 10 000 g for 5 min, washed with normal saline (0.9% NaCl) until the supernatant was clear, and resuspended using normal saline (100 mL) to prepare 2% erythrocyte solution. Then, different concentrations (0.5, 1, 2, 4, 8, 16, 32, 64, and 128 µg mL^−1^) of FL‐3 dissolved in normal saline solutions were added to the same‐volume 2% erythrocyte solution in centrifuge tubes. After incubation at 37 °C for 2 h, the supernatant was obtained through centrifugation at 10 000 g for 5 min, and transferred to a 96‐well plate. The absorbance at 540 nm was measured by a Multiskan FC microplate photometer (Thermo). RBCs in normal saline and in Deionized water were used as a negative control and a positive control, respectively. The following formula was used to calculate the hemolysis percentage:

Hemolysis (%) = (sample absorbance – negative control absorbance)/(positive control absorbance – negative control absorbance) × 100%.

### Cytotoxicity Assay

A549 were seeded in 96‐well plates for overnight culture at 37 °C, 5% CO_2_. Then the solution of FL‐3 was added into the cells and co‐cultured for 24 h. For the irradiation groups, the cells were cultured for another 24 h. Then, MTT solution (5 mg mL^−1^) was added into cells and incubated for another 4 h. Subsequently, DMSO (150 µL) was applied to replace the medium, and the absorbance solution was at 570 nm was measured by using a microplate reader and the cell viabilities were analyzed.

## Conflict of Interest

The authors declare no conflict of interest.

## Supporting information

Supporting Information

## Data Availability

The data that support the findings of this study are available from the corresponding author upon reasonable request.
