# Supplementary material for: AI‐Powered Mining of Highly Customized and Superior ESIPT‐Based Fluorescent Probes
Source: Adv Sci (Weinh). 2024 Jul 17;11(35):2405596. doi: 10.1002/advs.202405596 (PMC11425259; doi:10.1002/advs.202405596)

Supporting Information

AI-Powered Mining of Highly Customized and Superior ESIPT-Based Fluorescent Probes

Wenzhi Huang, Shuai Huang, Yanpeng Fang, Tianyu Zhu, Feiyi Chu, Qianhui Liu, Kunqian Yu, Fei Chen, Jie Dong^*^, Wenbin Zeng^*^

This file includes:

1. Supplementary materials for structure identification

2. Supplementary Tables S1 to S10

3. Supplementary Figures S1 to S14

**Supplementary materials for structure identification**

Instruments and measurements

1H-NMR and 13C-NMR were measured on a Bruker AVII-400 MHz or 500 MHz spectrometers with chemical shifts reported in ppm (in DMSO-d6 or CDCl_3_, TMS as internal standard). HRMS was obtained on an Orbitrap Velos Pro LC-MS spectrometer (Thermo Scientific, American). UV-vis absorption spectra were performed on a UV-2450 scanning spectrophotometer (Shimadzu, Japan). Fluorescent spectra were recorded on a Hitachi F-2700 equipped with a 1 cm quartz cell. Dynamic light scattering measurements were performed at 25 oC on Zestier Nano ZS (Malvern Instruments Ltd, UK).

**Compound synthesis**

**Synthetic route of Compound FL-3**


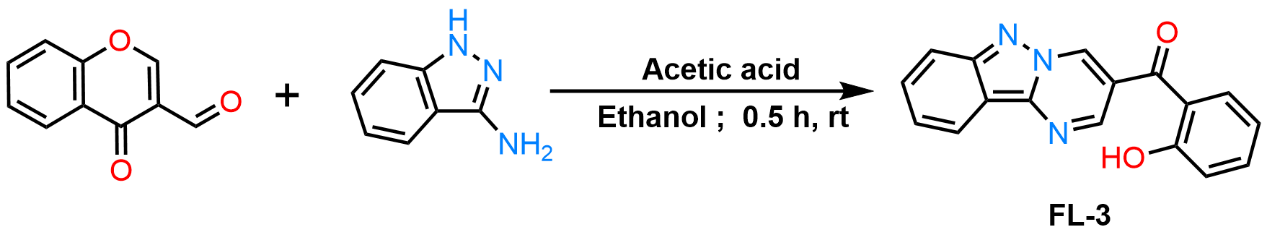


Synthesis of Compound FL-3: Dissolve 3-amino-1H-indazole (266 mg, 2 mM) and chromone-3-carboxaldehyde (348 mg, 2 mM) in ethanol respectively, add a small amount of acetic acid, and stir the system at room temperature for 6 h. After the reaction was completed, the pH was adjusted to neutral, extracted three times with DCM, the organic layers were combined, dried over anhydrous magnesium sulfate, and then separated and purified by silica gel column to obtain FL-3 (320 mg, 55%) ^1^H NMR (500 MHz, Chloroform-d) δ 11.60 (s, 1H), 9.37 (d, J = 2.1 Hz, 1H), 8.97 (d, J = 2.0 Hz, 1H), 8.37 (dt, J = 8.4, 1.1 Hz, 1H), 7.90 (dt, J = 8.7, 0.9 Hz, 1H), 7.75 – 7.66 (m, 2H), 7.62 (ddd, J = 8.7, 7.2, 1.7 Hz, 1H), 7.41 (ddd, J = 8.5, 6.7, 0.9 Hz, 1H), 7.17 (dd, J = 8.4, 1.1 Hz, 1H), 7.00 (ddd, J = 8.2, 7.2, 1.1 Hz, 1H). ^13^C NMR (126 MHz, Chloroform-d) δ 195.22, 163.30, 153.20, 144.82, 137.57, 135.54, 132.47, 131.18, 122.33, 121.03, 119.54, 119.13, 118.83, 116.66, 113.46. HR-MS (m/z): calculated for C17H12N3O2+ [M+H] +, 290.3015; found, 290.2704.

Table S1. The evaluation results of E-CM model.

| Descriptor | Algorithm | CV | | | | | Test | | | | |
| --- | --- | --- | --- | --- | --- | --- | --- | --- | --- | --- | --- |
|  |  | ACC | AUC | F1 | SP | SE | ACC | AUC | F1 | SP | SE |
| ECFP4 | RF | 0.904 | 0.962 | 0.907 | 0.927 | 0.881 | 0.902 | 0.963 | 0.904 | 0.902 | 0.902 |
|  | SVM | 0.887 | 0.950 | 0.891 | 0.860 | 0.914 | 0.887 | 0.958 | 0.890 | 0.867 | 0.906 |
|  | KNN | 0.853 | 0.931 | 0.861 | 0.800 | 0.906 | 0.845 | 0.933 | 0.859 | 0.760 | 0.927 |
|  | DT | 0.851 | 0.853 | 0.853 | 0.838 | 0.863 | 0.871 | 0.871 | 0.875 | 0.858 | 0.885 |
|  | XGBoost | 0.899 | 0.955 | 0.901 | 0.881 | 0.917 | 0.871 | 0.952 | 0.874 | 0.867 | 0.876 |
|  | DNN | 0.847 | 0.934 | 0.856 | 0.785 | 0.910 | 0.867 | 0.951 | 0.867 | 0.881 | 0.854 |
| MACCS | RF | 0.856 | 0.923 | 0.857 | 0.856 | 0.857 | 0.847 | 0.929 | 0.850 | 0.849 | 0.846 |
|  | SVM | 0.852 | 0.916 | 0.849 | 0.877 | 0.828 | 0.837 | 0.922 | 0.832 | 0.876 | 0.789 |
|  | KNN | 0.807 | 0.874 | 0.801 | 0.841 | 0.773 | 0.837 | 0.918 | 0.833 | 0.876 | 0.799 |
|  | DT | 0.833 | 0.834 | 0.831 | 0.848 | 0.818 | 0.830 | 0.828 | 0.830 | 0.849 | 0.812 |
|  | XGBoost | 0.864 | 0.920 | 0.862 | 0.875 | 0.853 | 0.863 | 0.918 | 0.861 | 0.893 | 0.833 |
|  | DNN | 0.833 | 0.897 | 0.817 | 0.918 | 0.750 | 0.850 | 0.914 | 0.845 | 0.898 | 0.803 |
| 2D | RF | 0.872 | 0.938 | 0.873 | 0.865 | 0.879 | 0.865 | 0.929 | 0.868 | 0.862 | 0.868 |
|  | SVM | 0.844 | 0.911 | 0.844 | 0.841 | 0.846 | 0.856 | 0.918 | 0.861 | 0.832 | 0.880 |
|  | KNN | 0.747 | 0.828 | 0.741 | 0.772 | 0.721 | 0.747 | 0.821 | 0.744 | 0.773 | 0.722 |
|  | DT | 0.792 | 0.792 | 0.793 | 0.789 | 0.796 | 0.778 | 0.778 | 0.782 | 0.773 | 0.782 |
|  | XGBoost | 0.879 | 0.940 | 0.869 | 0.870 | 0.868 | 0.852 | 0.928 | 0.855 | 0.849 | 0.855 |
|  | DNN | 0.783 | 0.885 | 0.759 | 0.883 | 0.683 | 0.808 | 0.892 | 0.817 | 0.773 | 0.842 |

Table S2. The evaluation results of E-FL model.

| Descriptor | Algorithm | CV | | | | | Test | | | | |
| --- | --- | --- | --- | --- | --- | --- | --- | --- | --- | --- | --- |
|  |  | ACC | AUC | F1 | SP | SE | ACC | AUC | F1 | SP | SE |
| ECFP4 | RF | 0.916 | 0.974 | 0.931 | 0.867 | 0.950 | 0.907 | 0.976 | 0.921 | 0.877 | 0.930 |
|  | SVM | 0.920 | 0.964 | 0.931 | 0.869 | 0.949 | 0.931 | 0.973 | 0.942 | 0.871 | 0.973 |
|  | KNN | 0.878 | 0.942 | 0.901 | 0.783 | 0.943 | 0.902 | 0.968 | 0.919 | 0.840 | 0.947 |
|  | DT | 0.889 | 0.889 | 0.907 | 0.862 | 0.907 | 0.910 | 0.914 | 0.922 | 0.907 | 0.912 |
|  | XGBoost | 0.920 | 0.971 | 0.933 | 0.883 | 0.946 | 0.910 | 0.970 | 0.923 | 0.883 | 0.930 |
|  | DNN | 0.875 | 0.937 | 0.901 | 0.742 | 0.965 | 0.889 | 0.952 | 0.911 | 0.809 | 0.944 |
| MACCS | RF | 0.905 | 0.966 | 0.921 | 0.856 | 0.938 | 0.913 | 0.968 | 0.926 | 0.855 | 0.955 |
|  | SVM | 0.900 | 0.958 | 0.917 | 0.858 | 0.930 | 0.933 | 0.979 | 0.941 | 0.922 | 0.941 |
|  | KNN | 0.827 | 0.913 | 0.860 | 0.726 | 0.896 | 0.884 | 0.947 | 0.904 | 0.795 | 0.951 |
|  | DT | 0.883 | 0.879 | 0.901 | 0.864 | 0.896 | 0.882 | 0.884 | 0.895 | 0.880 | 0.883 |
|  | XGBoost | 0.900 | 0.958 | 0.916 | 0.860 | 0.926 | 0.915 | 0.966 | 0.929 | 0.849 | 0.964 |
|  | DNN | 0.876 | 0.939 | 0.895 | 0.862 | 0.886 | 0.871 | 0.932 | 0.891 | 0.807 | 0.919 |
| 2D | RF | 0.899 | 0.958 | 0.917 | 0.821 | 0.952 | 0.905 | 0.961 | 0.921 | 0.819 | 0.969 |
|  | SVM | 0.889 | 0.944 | 0.910 | 0.817 | 0.939 | 0.902 | 0.958 | 0.917 | 0.838 | 0.950 |
|  | KNN | 0.799 | 0.857 | 0.714 | 0.602 | 0.928 | 0.789 | 0.866 | 0.835 | 0.619 | 0.922 |
|  | DT | 0.839 | 0.842 | 0.861 | 0.837 | 0.841 | 0.871 | 0.869 | 0.888 | 0.843 | 0.892 |
|  | XGBoost | 0.913 | 0.958 | 0.926 | 0.855 | 0.955 | 0.909 | 0.958 | 0.924 | 0.861 | 0.941 |
|  | DNN | 0.871 | 0.939 | 0.891 | 0.843 | 0.890 | 0.889 | 0.937 | 0.908 | 0.908 | 0.955 |

Table S3. The evaluation results of E-Barrier model.

| Descriptor | Metric | RF | | SVM | | XGBoost | |
| --- | --- | --- | --- | --- | --- | --- | --- |
|  |  | CV | Test | CV | Test | CV | Test |
| 2D+3D | R^2^ | 0.429 | 0.384 | 0.397 | 0.392 | 0.422 | 0.382 |
|  | MAE | 0.449 | 0.463 | 0.433 | 0.450 | 0.444 | 0.457 |
|  | RMSE | 0.548 | 0.568 | 0.563 | 0.565 | 0.569 | 0.551 |
|  | Correlation | 0.660 | 0.626 | 0.633 | 0.627 | 0.651 | 0.627 |
|  | 2-fold rate | 0.753 | 0.749 | 0.760 | 0.759 | 0.749 | 0.728 |
|  | 3-fold rate | 0.795 | 0.821 | 0.803 | 0.821 | 0.801 | 0.809 |

Table S4. Scoring metrics for toxicity and safety strategy.

| Toxicity and Safety | | Range | Score |
| --- | --- | --- | --- |
| Important Descriptor | BCUT_PEOE_2 | 0.619 <= BCUT_PEOE_2 <= 0.704 | +1 |
|  |  | Else | 0 |
|  | GCUT_SMR_2 | 0.193 <= GCUT_SMR_2 <= 0.283 | +1 |
|  |  | Else | 0 |
|  | SlogP_VSA0 | 25.3 <= SlogP_VSA0 <= 36.4 | +1 |
|  |  | Else | 0 |
|  | a_nO | 1 <= a_nO <= 3 | +1 |
|  |  | Else | 0 |
|  | h_pKa | 7.09 <= h_pKa <= 9.47 | +1 |
|  |  | Else | 0 |
|  | h_pavgQ | -0.445 <= h_pavgQ <= 0 | +1 |
|  |  | Else | 0 |
|  | h_pstrain | 0.0866 <= h_pstrain <= 2.03 | +1 |
|  |  | Else | 0 |
| Physicochemical properties | TPSA | 0 <= TPSA < 140 | +1 |
|  |  | Else | 0 |
|  | nHA | 0 < nHA <= 12 | +1 |
|  |  | Else | 0 |
|  | nHD | 0 <= nHD <= 7 | +1 |
|  |  | Else | 0 |
|  | nRing | 0 <= nRing <= 6 | +1 |
|  |  | Else | 0 |
|  | nRot | 1 <= nRot <= 11 | +1 |
|  |  | Else | 0 |
|  | nRig | 0 <= nRig <= 30 | +1 |
|  |  | Else | 0 |
|  | maxRing | 0 <= maxRing <= 18 | +1 |
|  |  | Else | 0 |
|  | ROA | 0 <= ROA <= 0.3 | +1 |
|  |  | 0.3 < ROA <= 0.7 | +0.5 |
|  |  | Else | 0 |
| Toxicity | h_ht | 0 <= h_ht <= 0.3 | +1 |
|  |  | 0.3 < h_ht <= 0.7 | +0.5 |
|  |  | Else | 0 |
|  | Ames | 0 <= Ames <= 0.3 | +1 |
|  |  | 0.3 < Ames <= 0.7 | +0.5 |
|  |  | Else | 0 |
|  | hERG | 0 <= hERG <= 0.3 | +1 |
|  |  | 0.3 < hERG <= 0.7 | +0.5 |
|  |  | Else | 0 |
|  | SkinSen | 0 <= SkinSen <= 0.3 | +1 |
|  |  | 0.3 < SkinSen <= 0.7 | +0.5 |
|  |  | Else | 0 |
|  | LD50_oral | 0 <= LD50_oral <= 0.3 | +1 |
|  |  | 0.3 < LD50_oral <= 0.7 | +0.5 |
|  |  | Else | 0 |
|  | Respiratory | 0 <= Respiratory <= 0.3 | +1 |
|  |  | 0.3 < Respiratory <= 0.7 | +0.5 |
|  |  | Else | 0 |

Table S5. Scoring metrics for structural innovation strategy.

| Structural innovation | | Range | Score |
| --- | --- | --- | --- |
| Important Descriptor | BCUT_PEOE_2 | 0.619 <= BCUT_PEOE_2 <= 0.704 | +1 |
|  |  | Else | 0 |
|  | GCUT_SMR_2 | 0.193 <= GCUT_SMR_2 <= 0.283 | +1 |
|  |  | Else | 0 |
|  | SlogP_VSA0 | 25.3 <= SlogP_VSA0 <= 36.4 | +1 |
|  |  | Else |  |
|  | a_nO | 1 <= a_nO <= 3 | +1 |
|  |  | Else | 0 |
|  | h_pKa | 7.09 <= h_pKa <= 9.47 | +1 |
|  |  | Else | 0 |
|  | h_pavgQ | -0.445 <= h_pavgQ <= 0 | +1 |
|  |  | Else | 0 |
|  | h_pstrain | 0.0866 <= h_pstrain <= 2.03 | +1 |
|  |  | Else | 0 |
| Physicochemical properties | TPSA | 0 <= TPSA < 140 | +1 |
|  |  | Else | 0 |
|  | nHA | 0 < nHA <= 12 | +1 |
|  |  | Else | 0 |
|  | nHD | 0 <= nHD <= 7 | +1 |
|  |  | Else | 0 |
|  | nRing | 0 <= nRing <= 6 | +1 |
|  |  | Else | 0 |
|  | nRot | 1 <= nRot <= 11 | +1 |
|  |  | Else | 0 |
|  | nRig | 0 <= nRig <= 30 | +1 |
|  |  | Else | 0 |
|  | maxRing | 0 <= maxRing <= 18 | +1 |
|  |  | Else | 0 |
|  | ROA | 0 <= ROA <= 0.3 | +1 |
|  |  | 0.3 < ROA <= 0.7 | +0.5 |
|  |  | Else | 0 |

Table S6. Scoring metrics for pharmacokinetics strategy.

| Pharmacokinetics | | Range | Score |
| --- | --- | --- | --- |
| Important Descriptor | BCUT_PEOE_2 | 0.619 <= BCUT_PEOE_2 <= 0.704 | +1 |
|  |  | Else | 0 |
|  | GCUT_SMR_2 | 0.193 <= GCUT_SMR_2 <= 0.283 | +1 |
|  |  | Else | 0 |
|  | SlogP_VSA0 | 25.3 <= SlogP_VSA0 <= 36.4 | +1 |
|  |  | Else |  |
|  | a_nO | 1 <= a_nO <= 3 | +1 |
|  |  | Else | 0 |
|  | h_pKa | 7.09 <= h_pKa <= 9.47 | +1 |
|  |  | Else | 0 |
|  | h_pavgQ | -0.445 <= h_pavgQ <= 0 | +1 |
|  |  | Else | 0 |
|  | h_pstrain | 0.0866 <= h_pstrain <= 2.03 | +1 |
|  |  | Else | 0 |
| Physicochemical properties | TPSA | 0 <= TPSA < 140 | +1 |
|  |  | Else | 0 |
|  | nHA | 0 < nHA <= 12 | +1 |
|  |  | Else | 0 |
|  | nHD | 0 <= nHD <= 7 | +1 |
|  |  | Else | 0 |
|  | nRing | 0 <= nRing <= 6 | +1 |
|  |  | Else | 0 |
|  | nRot | 1 <= nRot <= 11 | +1 |
|  |  | Else | 0 |
|  | nRig | 0 <= nRig <= 30 | +1 |
|  |  | Else | 0 |
|  | maxRing | 0 <= maxRing <= 18 | +1 |
|  |  | Else | 0 |
|  | ROA | 0 <= ROA <= 0.3 | +1 |
|  |  | 0.3 < ROA <= 0.7 | +0.5 |
|  |  | Else | 0 |
| Pharmacokinetics | LogD | 1 <= LogD <= 3 | +1 |
|  |  | Else | 0 |
|  | T12 | 0 < T12 <= 0.3 | +1 |
|  |  | 0.3 < T12 <= 0.7 | +0.5 |
|  |  | Else | 0 |
|  | PPB | 0 < PPB <= 90 | +1 |
|  |  | Else | 0 |
|  | CL | 5 <= CL | +1 |
|  |  | Else | 0 |
|  | VDss | 0.04 <= VDss <= 20 | +1 |
|  |  | Else | 0 |
|  | Fu | Fu <= 5 | +1 |
|  |  | Else | 0 |

Table S7. The top 100 compounds in toxicity and safety strategy.


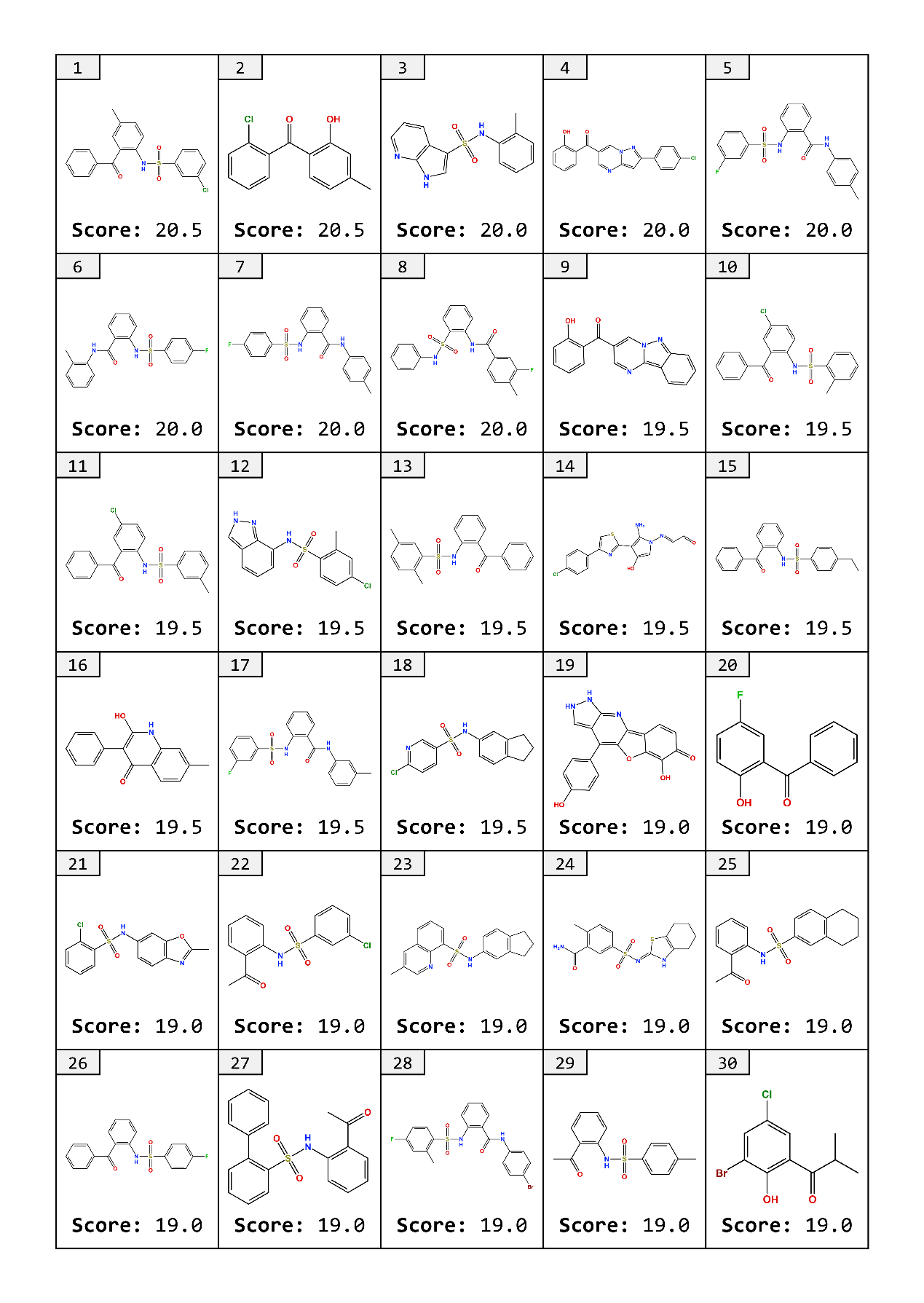


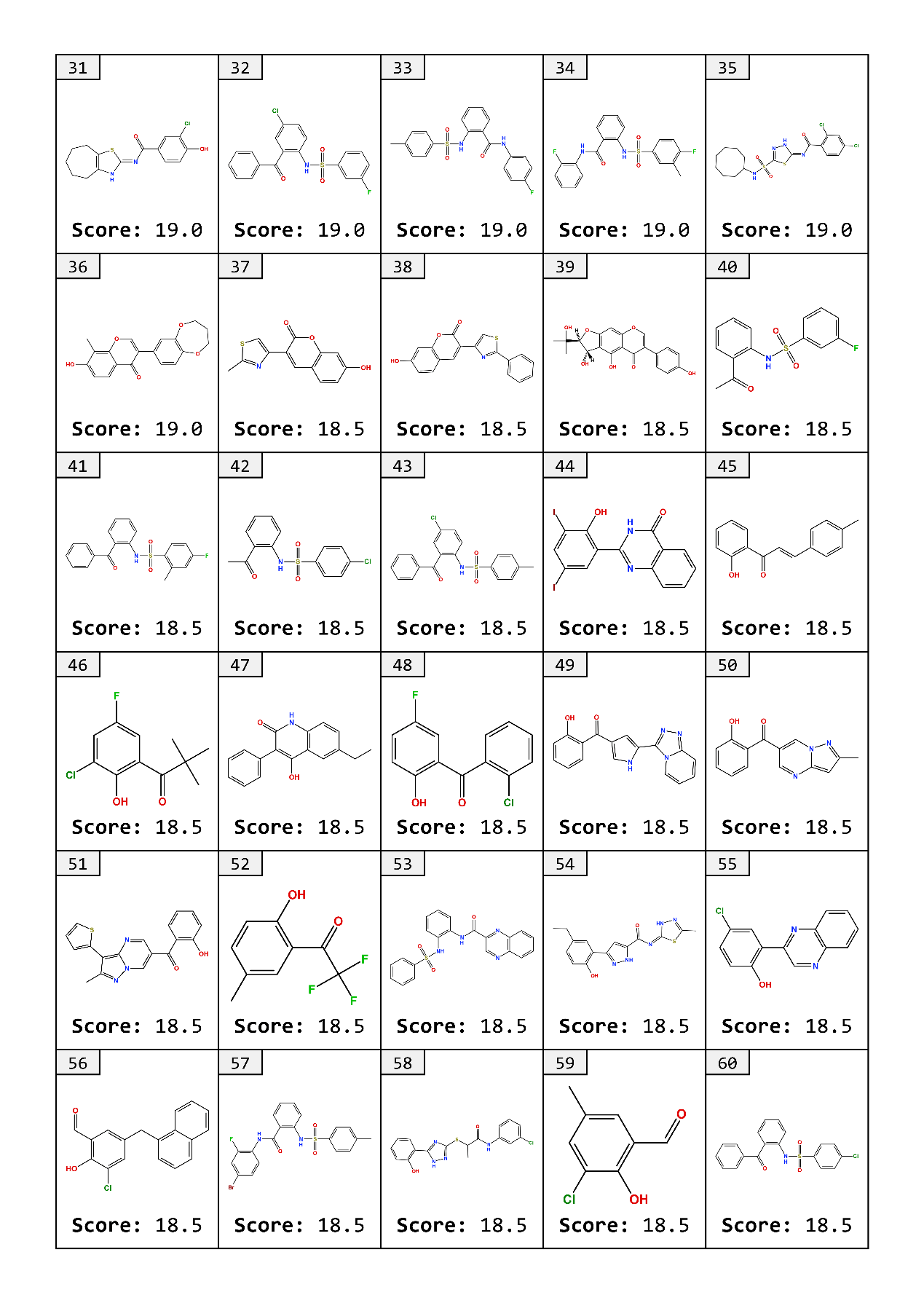

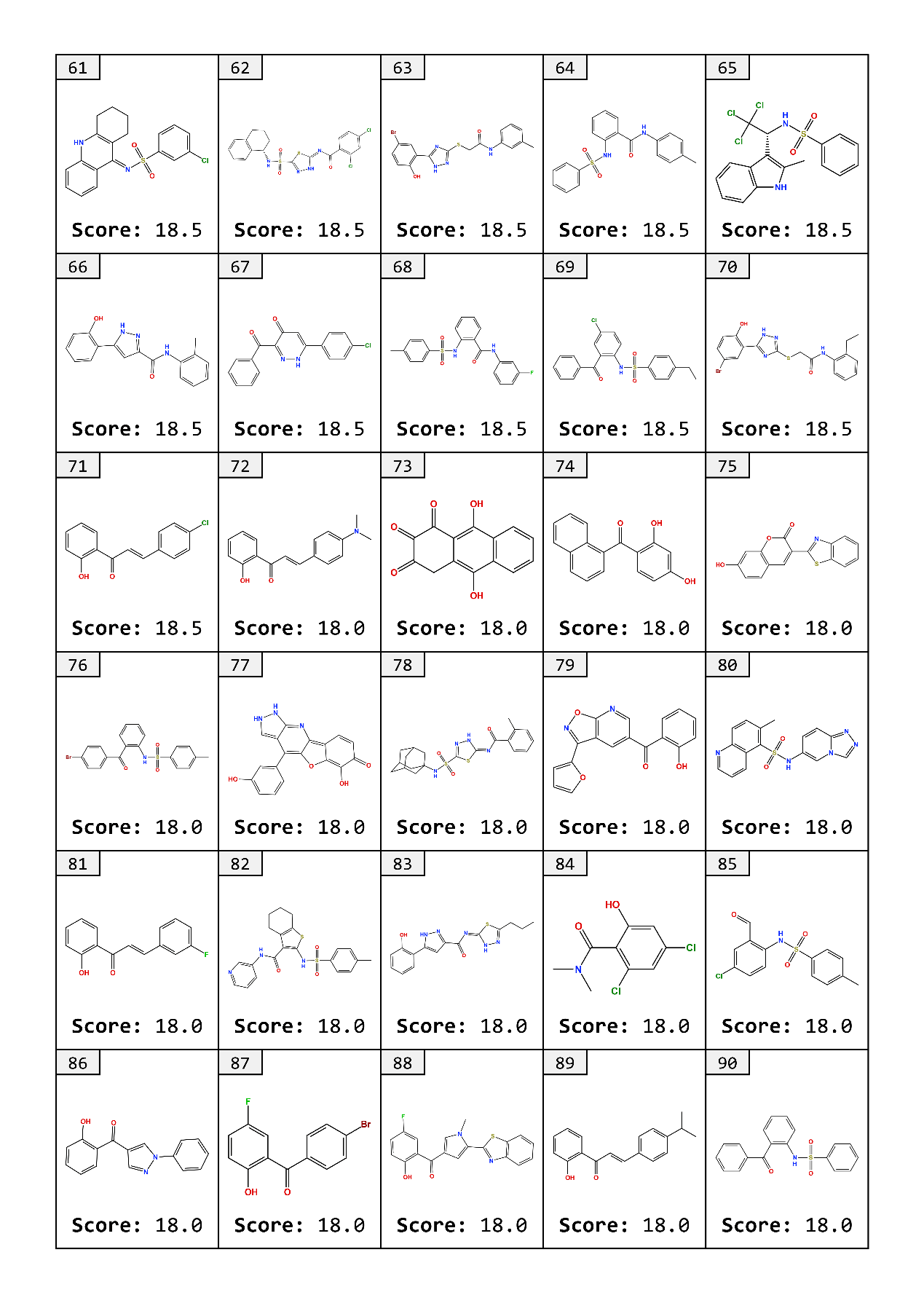


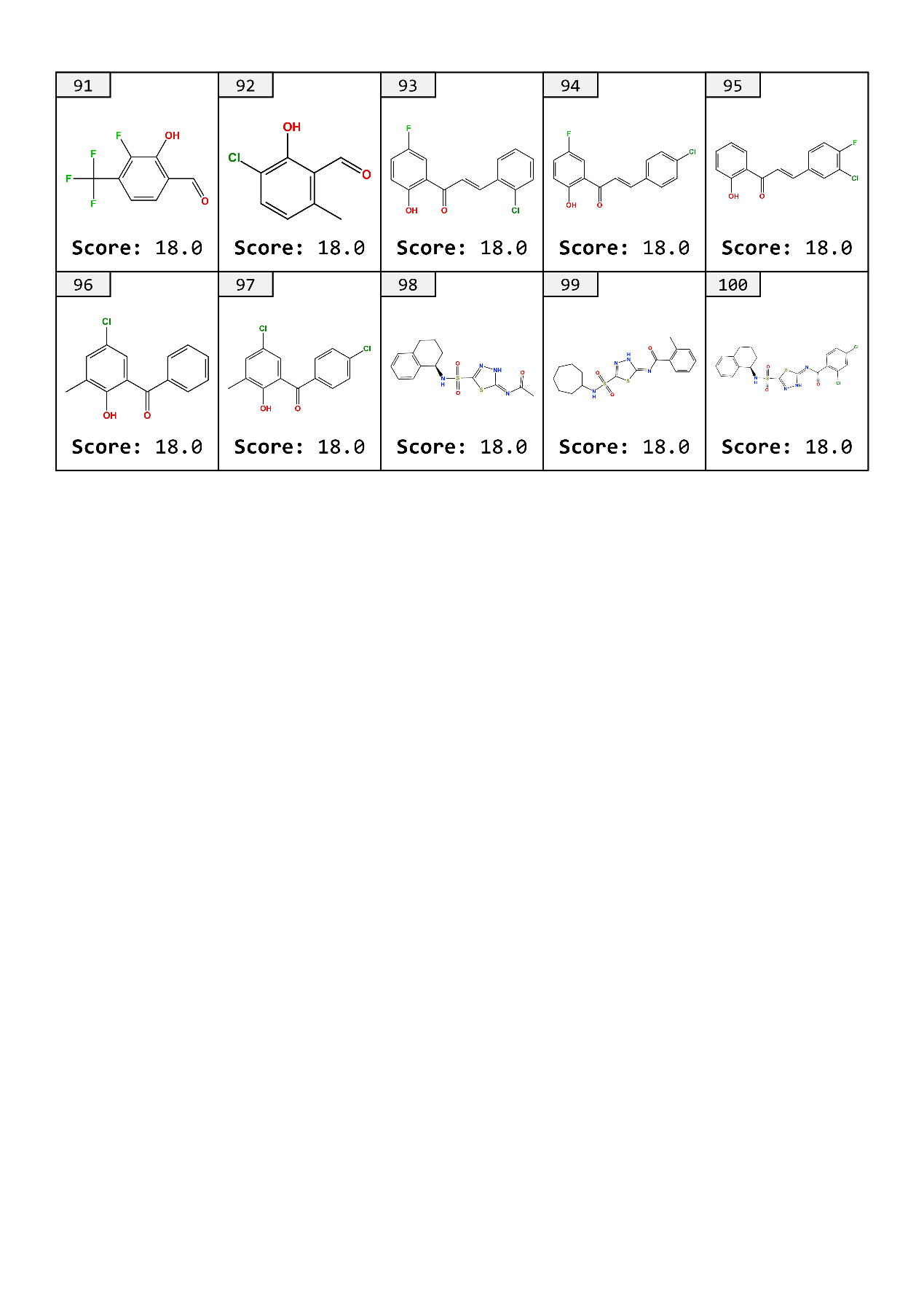


Table S8. The top 100 compounds in structural innovation strategy.


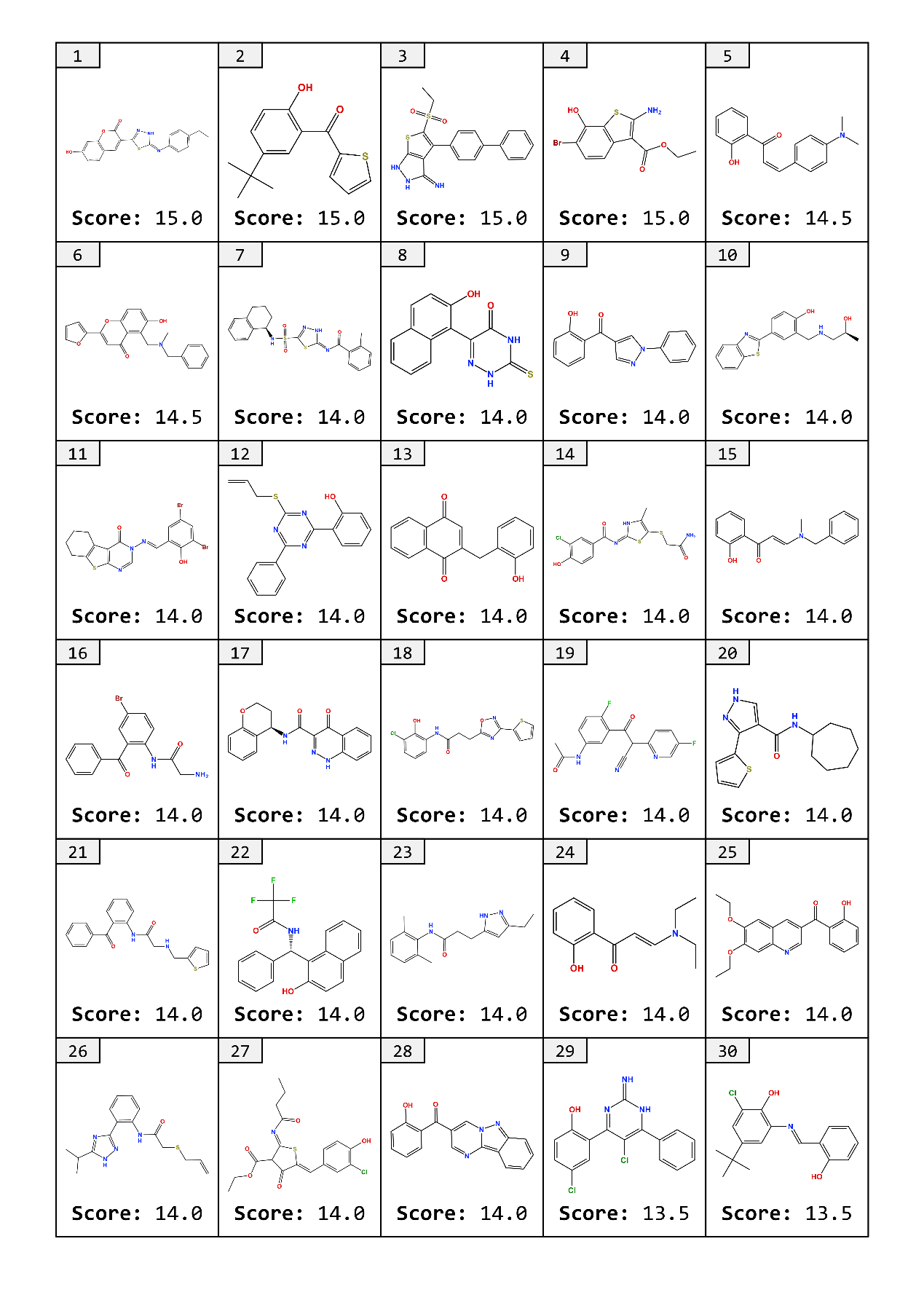


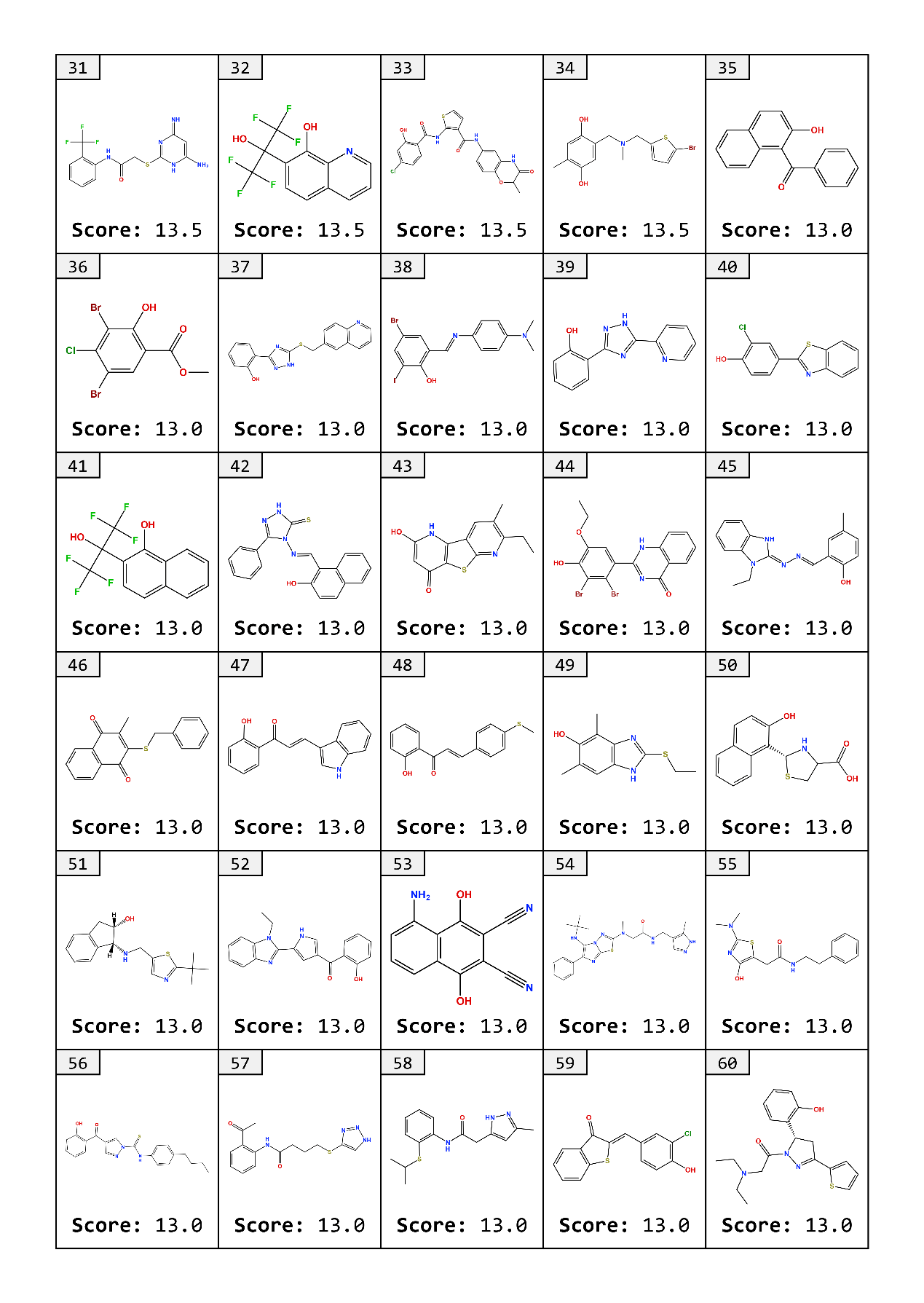


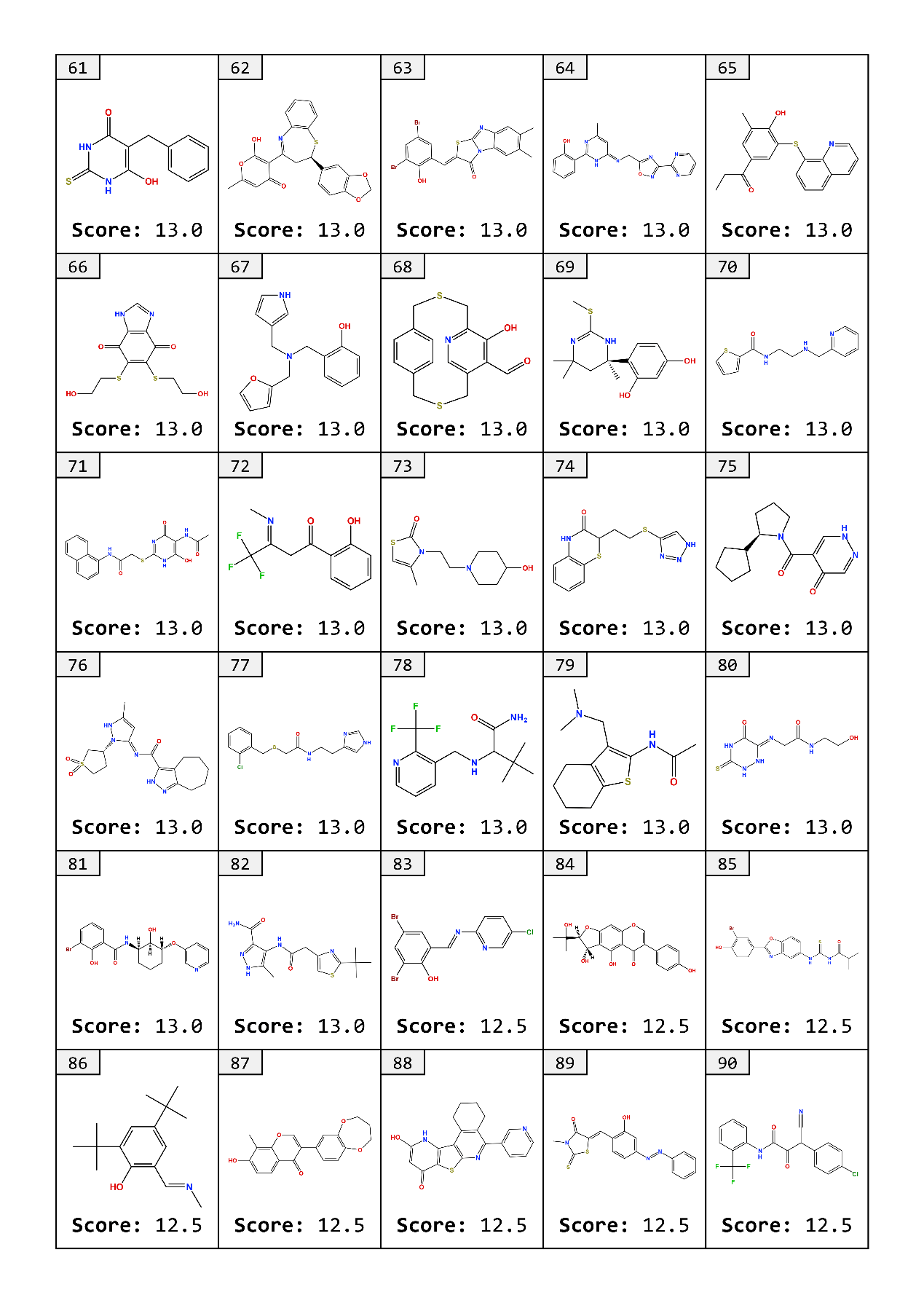


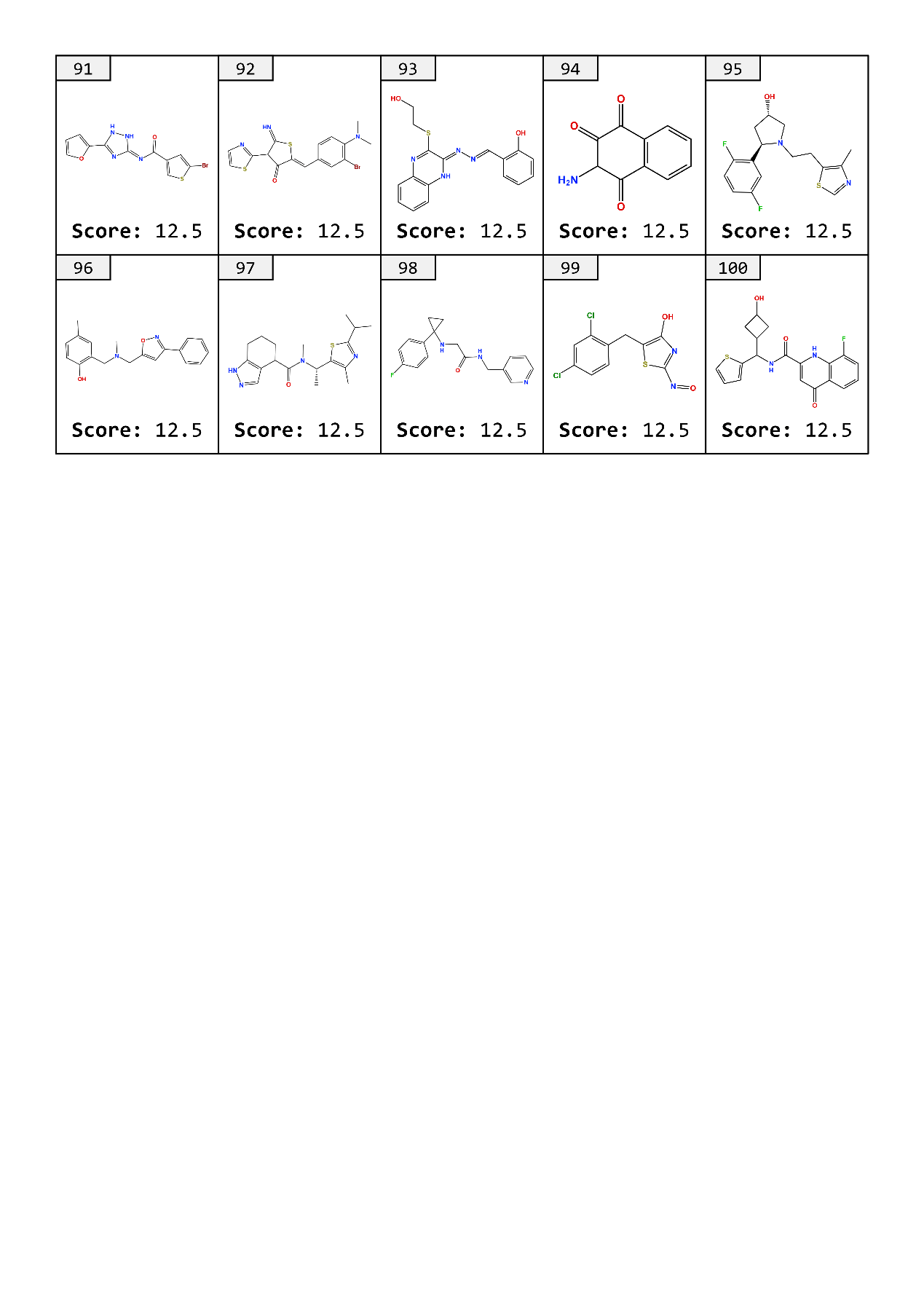


Table S9. The top 100 compounds in pharmacokinetics strategy.


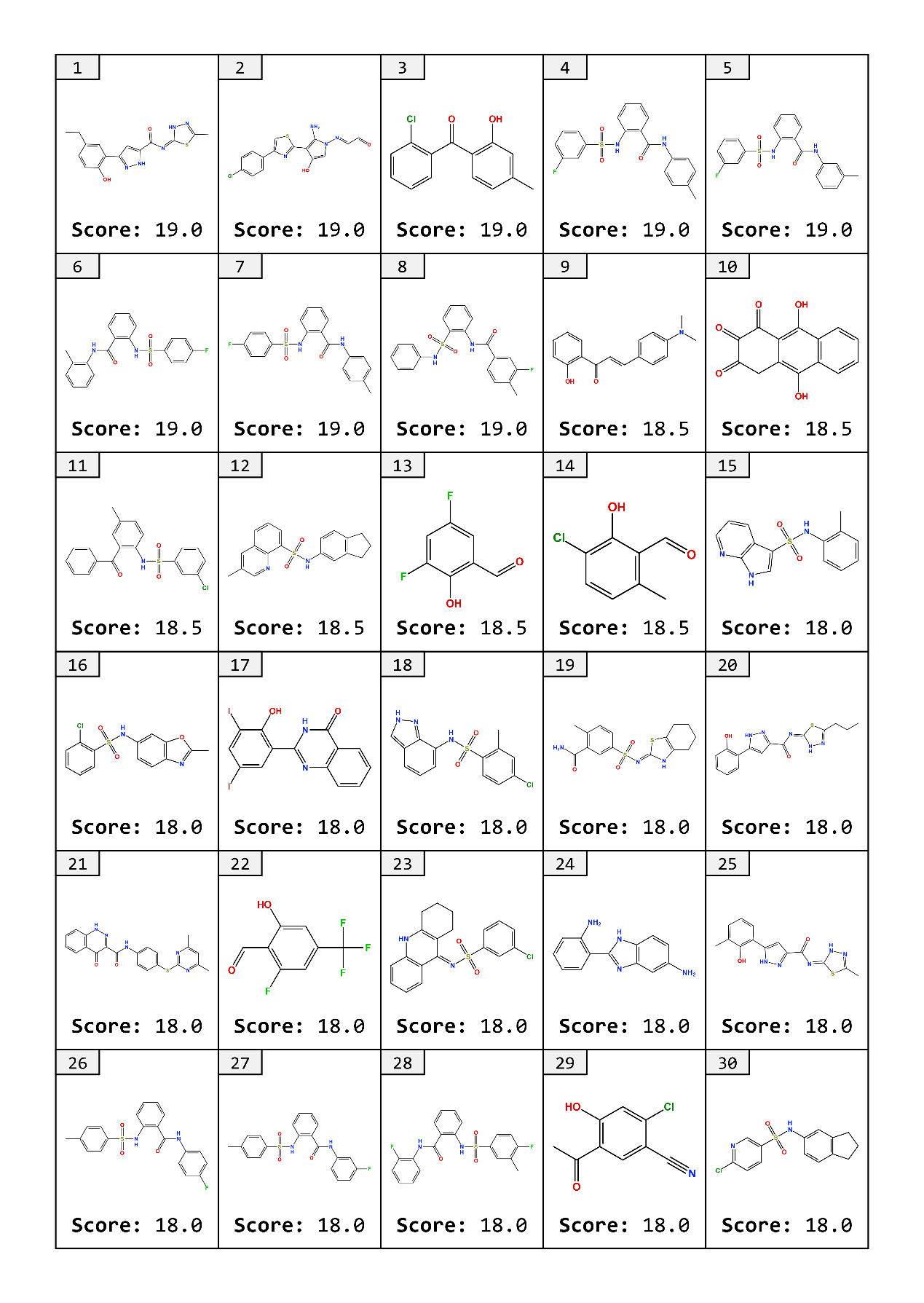


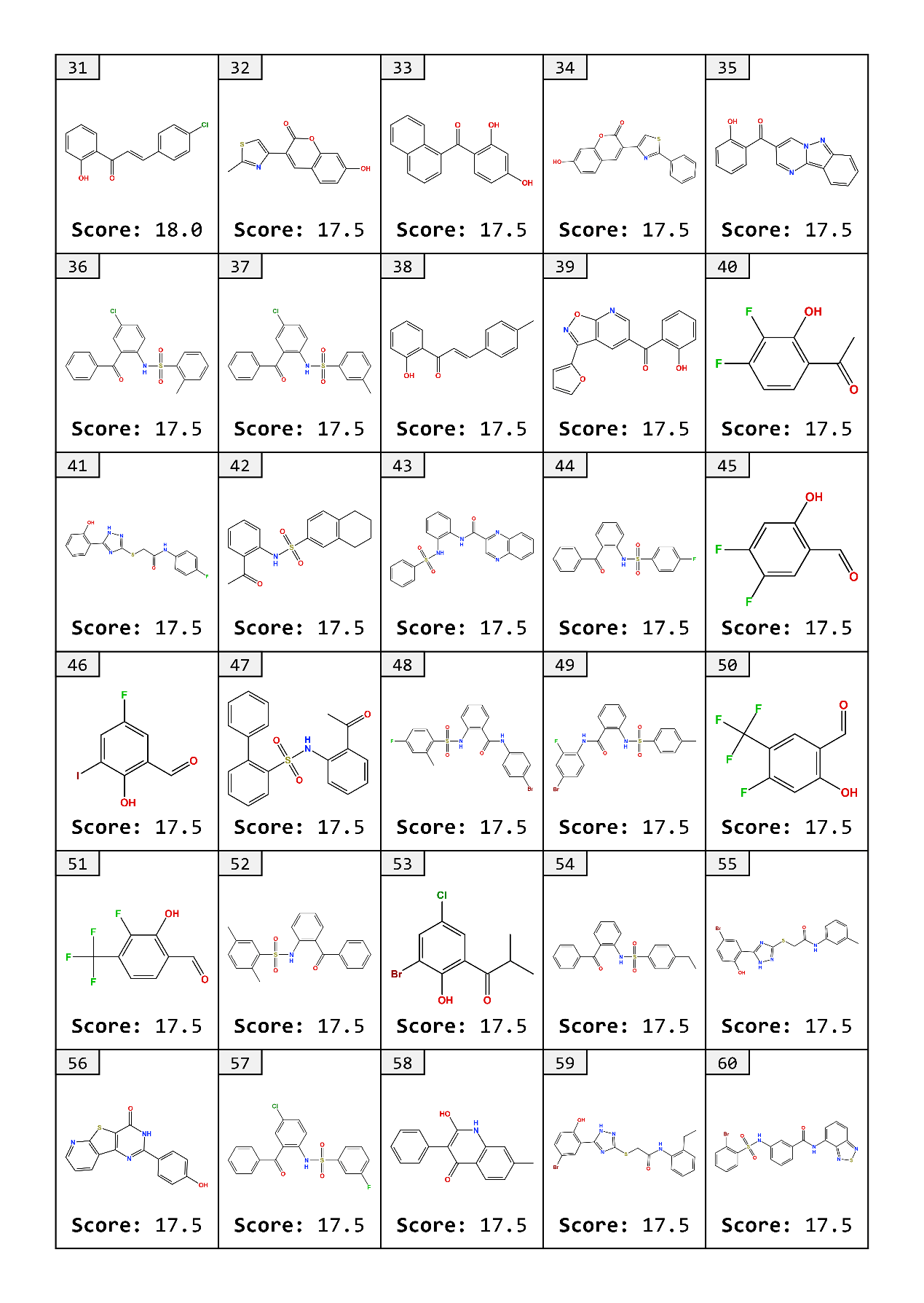

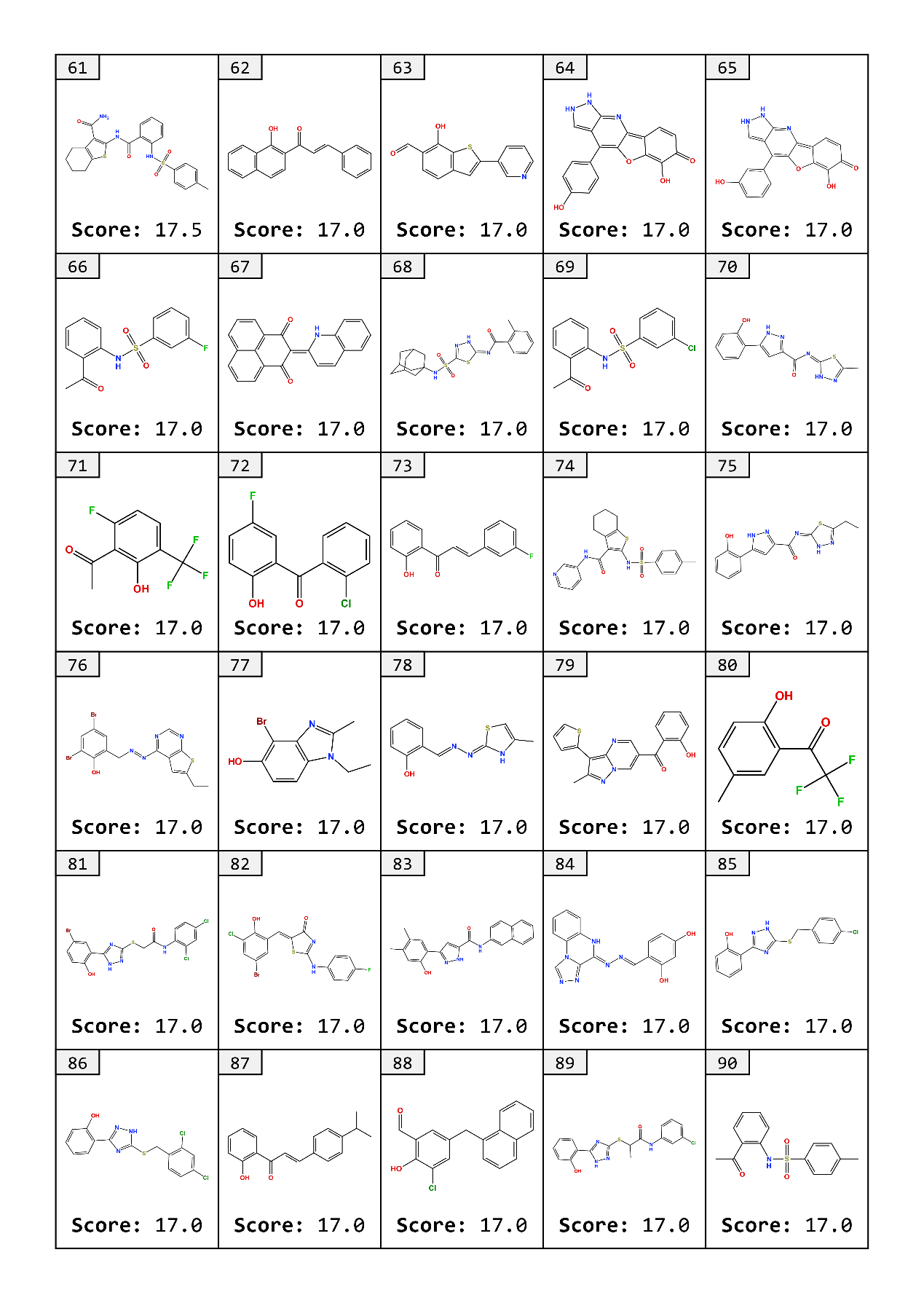


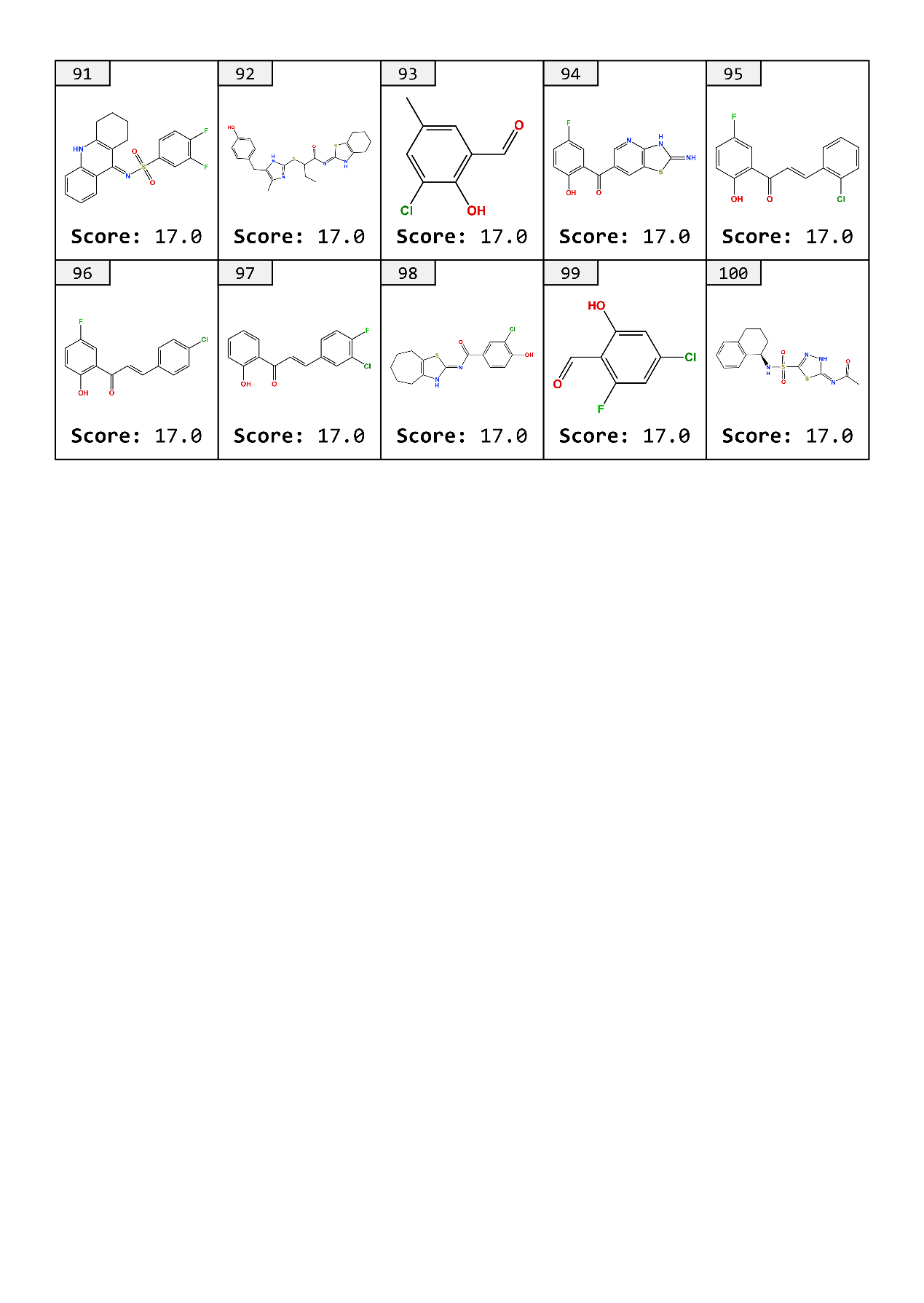


Table S10. Ten reported ESIPT molecules in our group.

| Ten reported ESIPT molecules | DOI |
| --- | --- |
| CC1=CC=C(S(=O)(=O)NC2=C(C3=NC4=CC=CC=C4S3)C=C(Cl)C=C2)C=C1 | 10.1016/j.cej.2023.146436 |
| CC1=CC=C(S(=O)(=O)NC2=C(C3=NC4=CC=CC=C4S3)C=CC=C2)C=C1 |  |
| CC1=CC=C(S(=O)(=O)NC2=C(C3=NC4=CC=CC=C4S3)C=C(N3C(=O)CCC3=O)C=C2)C=C1 | 10.1039/D1CC00429H |
| CC(=O)NC1=CC(C2=NC3=CC=CC=C3S2)=C(NS(=O)(=O)C2=CC=C(C)C=C2)C=C1 | 10.1016/j.snb.2023.134541 |
| CC1=CC=C(S(=O)(=O)NC2=C(C3=NC4=CC=CC=C4S3)C=C(NC(=O)C3=C(C(=O)O)C=CC=C3)C=C2)C=C1 | 10.1016/j.talanta.2018.09.104 |
| COC1=CC=C(C2=C(C3=CC=C(OC)C=C3)N(C3=CC=CC=C3)C(C3=CC=C(O)C(C4=NC5=C(C=CC=C5)S4)=C3)=N2)C=C1 | 10.1016/j.cclet.2022.107940 |
| OC1=C(C2=NC3=CC=CC=C3S2)C=C(C2=CC=C(N(C3=CC=CC=C3)C3=CC=CC=C3)C=C2)C=C1 | 10.1016/j.cclet.2021.10.024 |
| CC1=CC(/C=C(/C#N)C2=CC=C(C3=CC=[N+](CC4=CC=CC=C4)C=C3)C=C2)=CC(C2=NC3=CC=CC=C3S2)=C1O.[Br-] | 10.1016/j.cclet.2023.108273 |
| C[N+]1=CC=C(/C=C/C2=CC(C3=NC4=CC=CC=C4S3)=C(O)C=C2)C=C1.[I-] | 10.1002/asia.201500114 |
| OC1=C(C2=NC3=C(C=CC=C3)S2)C=C(C2=NC(C3=CC=CC=C3)=C(C3=CC=CC=C3)N2C2=CC=CC=C2)C=C1 | 10.1016/j.talanta.2020.121950 |

Table S11. The absorption and fluorescence spectra of compound FL-3 in different solvents.

| Compound |  | EA | ACN | THF | DMSO | MeOH | EtOH |
| --- | --- | --- | --- | --- | --- | --- | --- |
| FL-3 | λ_abs_(nm) | 335 | 335 | 335 | 335 | 335 | 336 |
|  | λ_Flu_ enol (nm) | - | 396 | 402 | - | - | - |
|  | λ_Flu_ keto (nm) | 525 | - | 548 | 537 | - | - |

Figure S1. (A) Classification report of the best E-CM model, (B) prediction error plots of the best E-CM model (Class 0: Positive; Class 1: Negative).


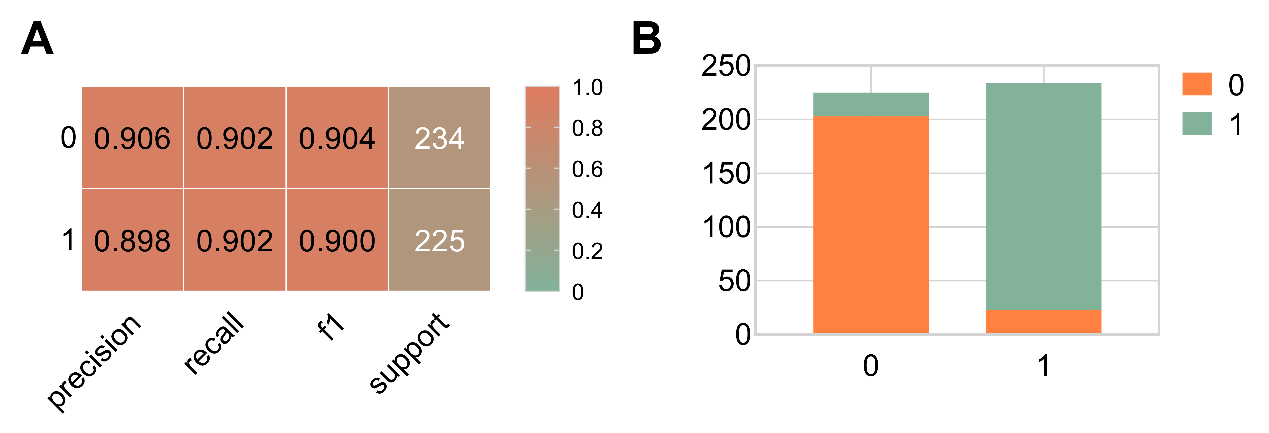


Figure S2. (A) Classification report of the best E-FL model, (B) prediction error plots of the best E-FL model (Class 0: Positive; Class 1: Negative).


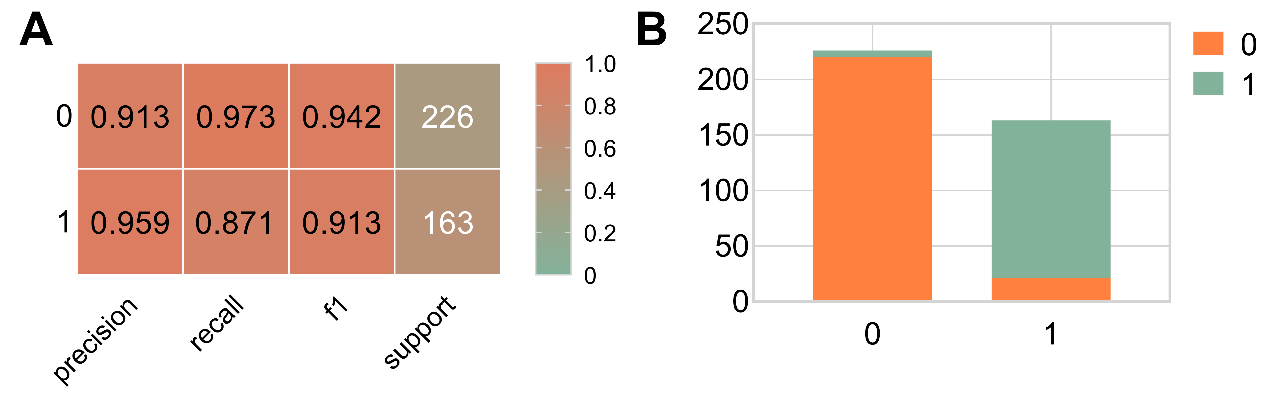


Figure S3. Plot of predicted value versus experimental of the E-Barrier model by SVM.


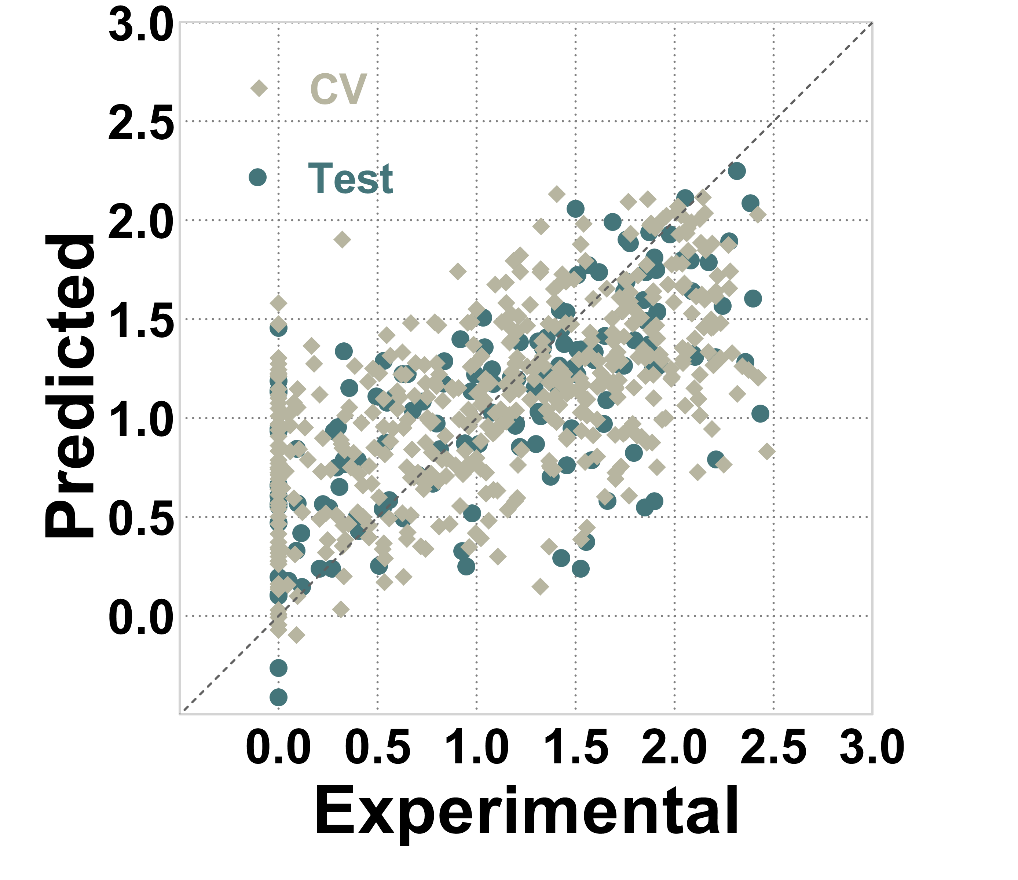


Figure S4. Plot of predicted value versus experimental of the E-Barrier model by XGBoost.


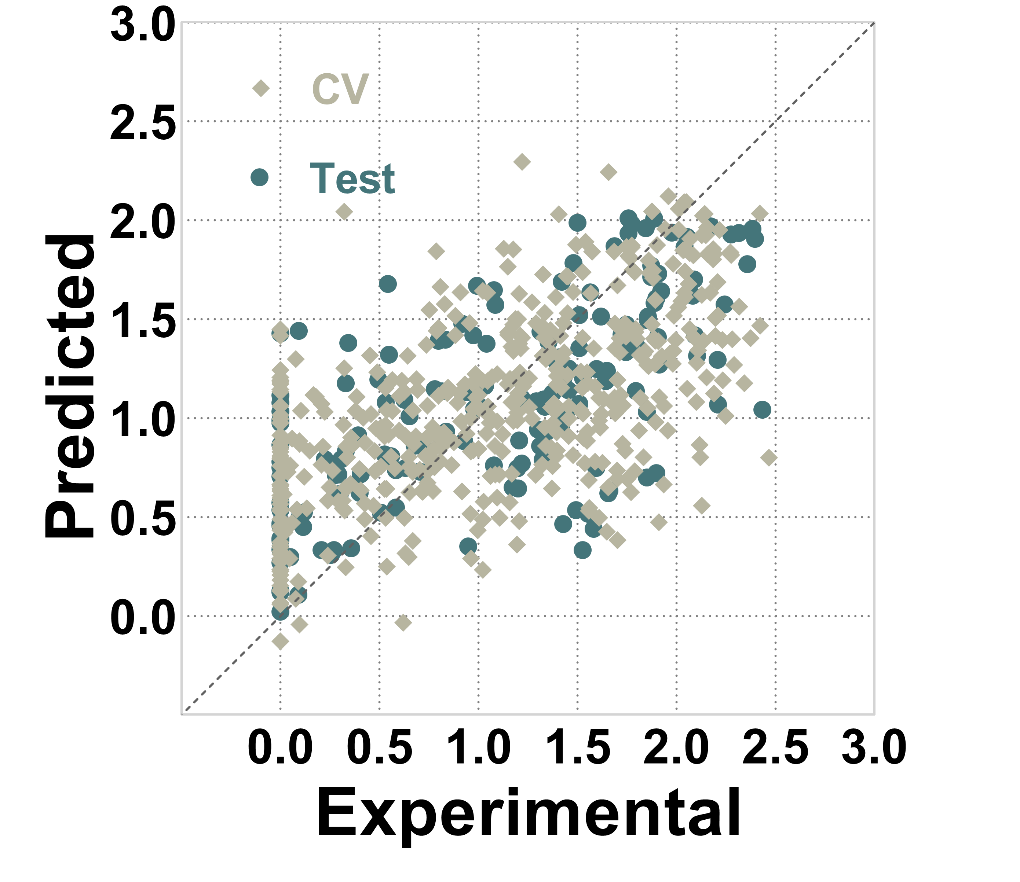


Figure S5. Fold rate plot for the E-Barrier model employing SVM.


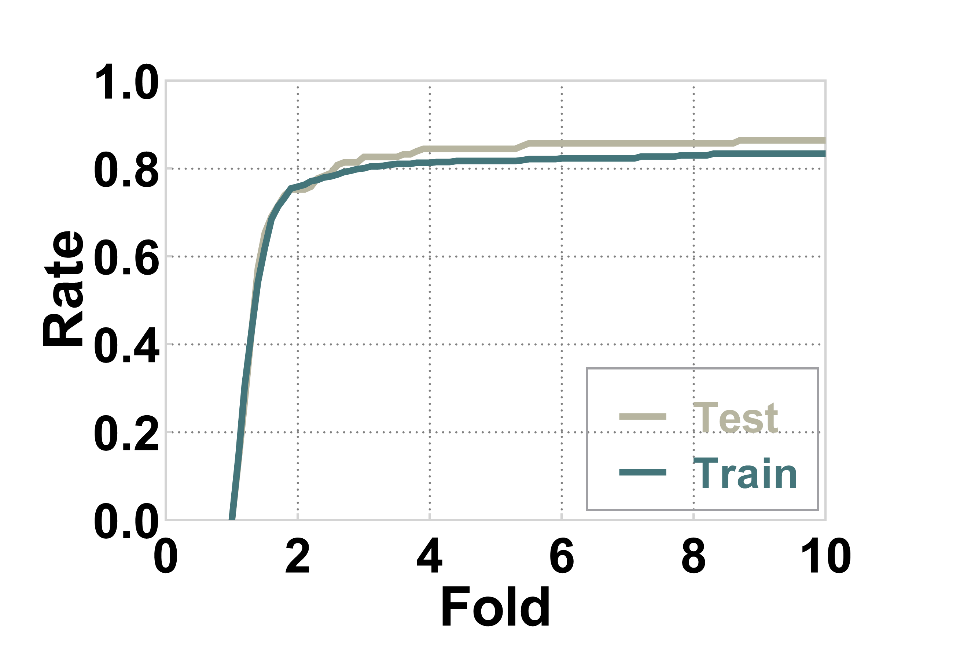


Figure S6. Fold rate plot for the E-Barrier model employing XGBoost.


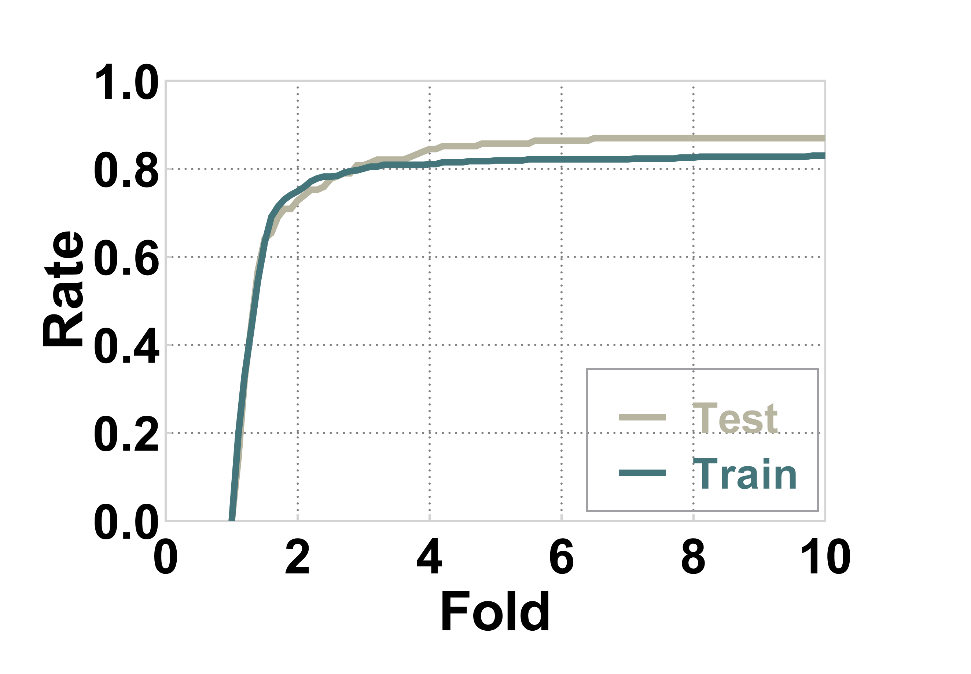


Figure S7. The average scores of the 10 ESIPT molecules reported within our group in three evaluation strategies, T: Toxicity and Safety, S: Structural Innovation, P: Pharmacokinetics.


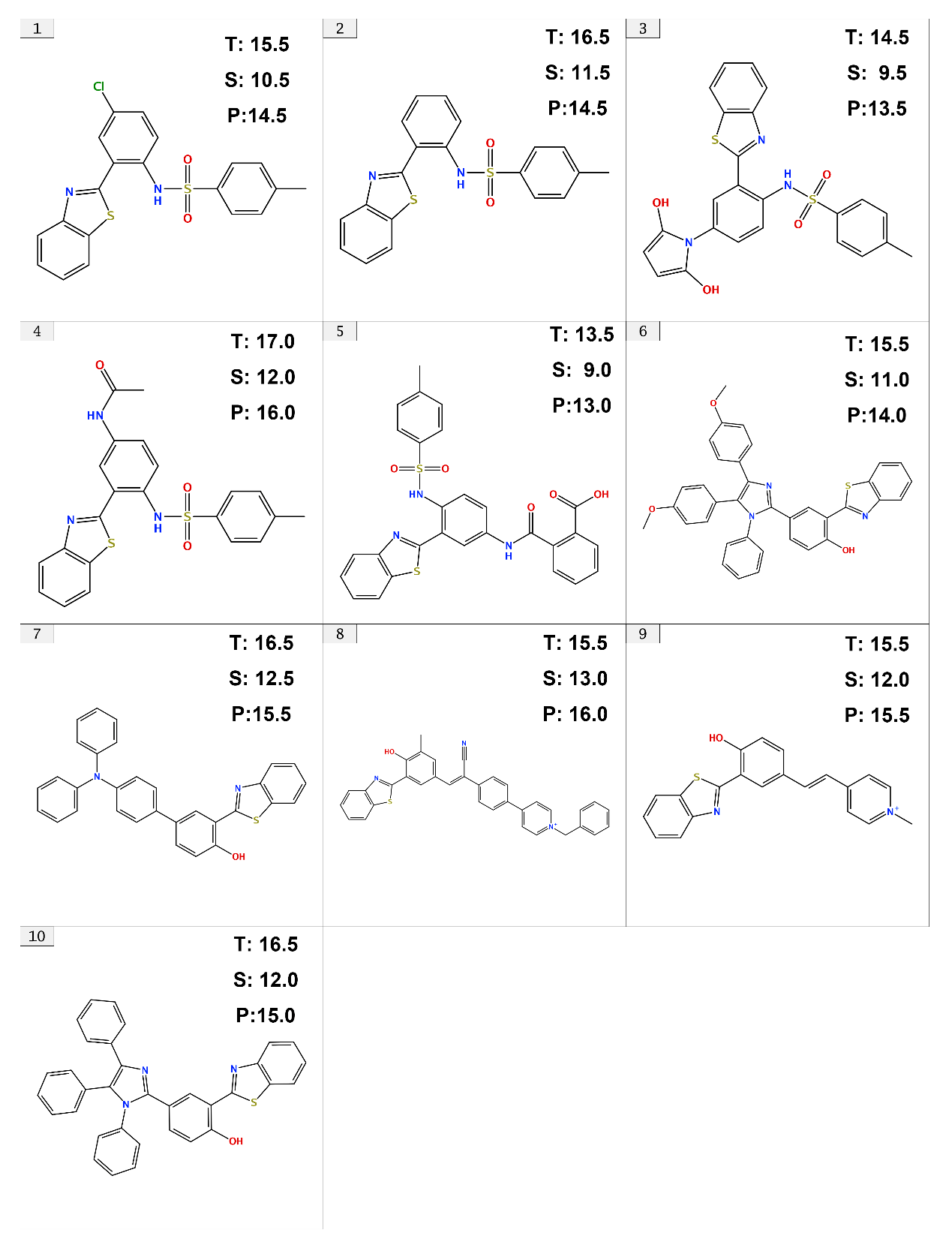


Figure S8. ^1^H-NMR spectrum of FL-3 in CDCl_3_.

**
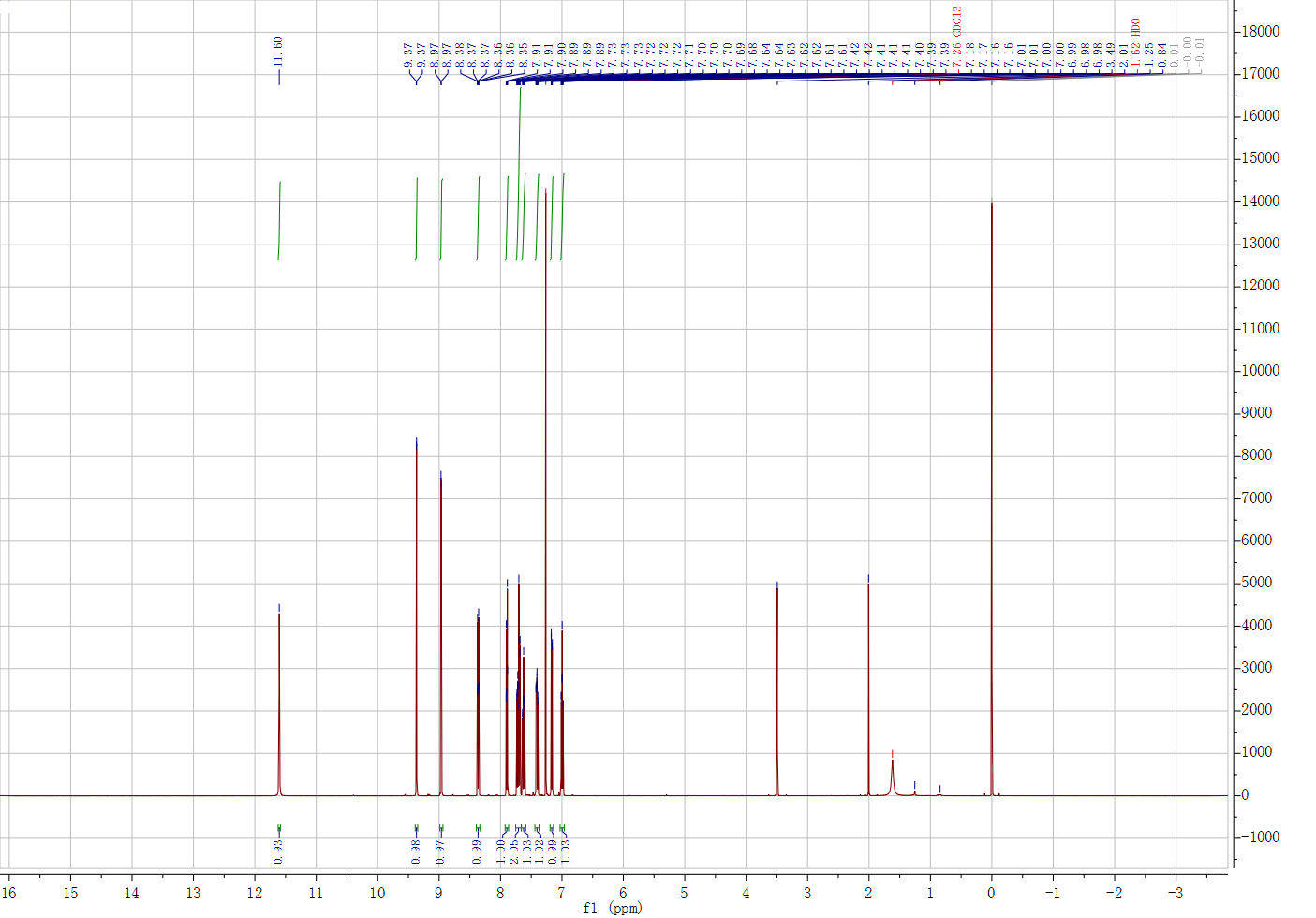
**

Figure S9. ^3^C-NMR spectrum of FL-3 in CDCl_3_.

**
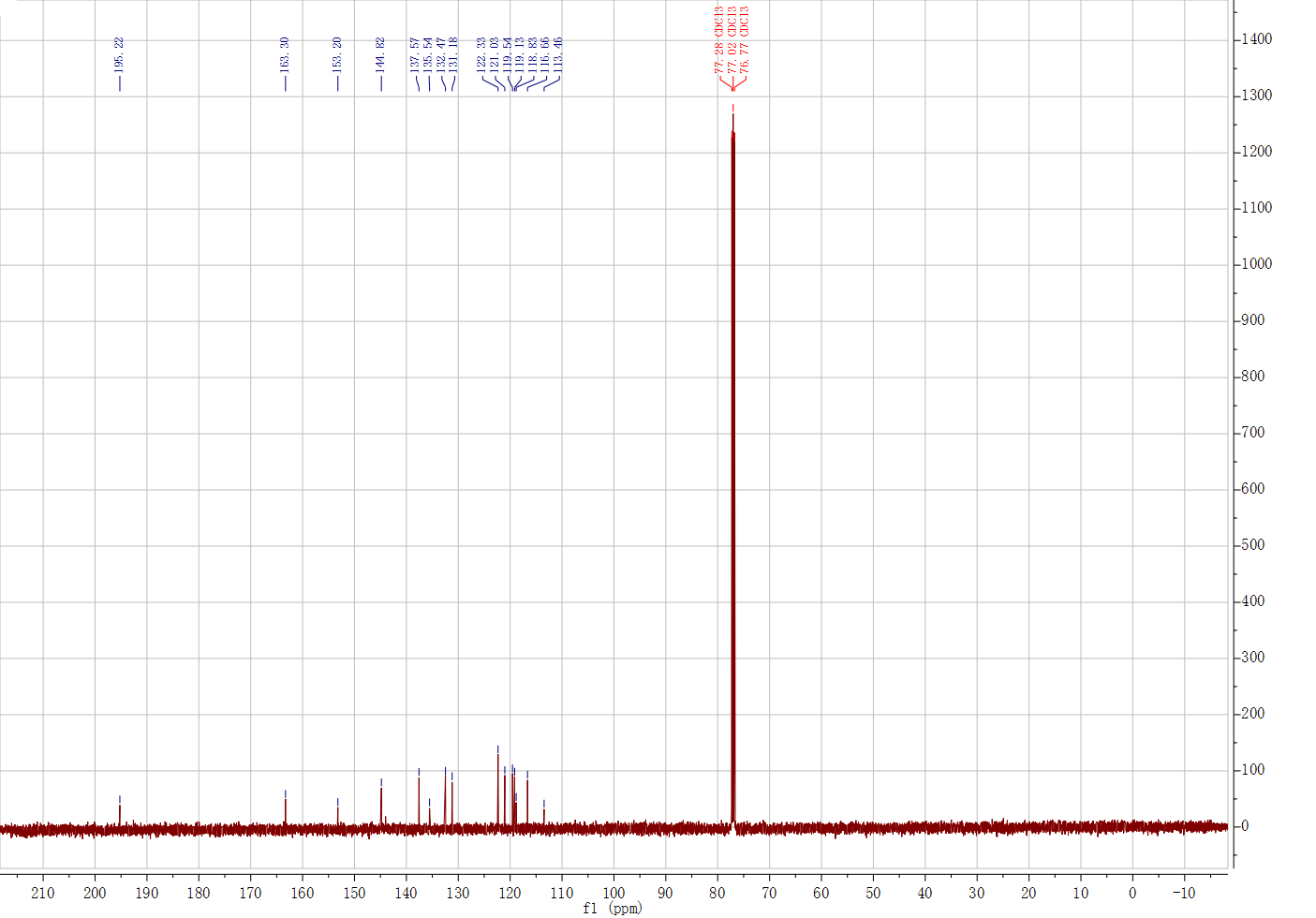
**

**Figure S10. HR-MS spectrum of compound FL-3.**


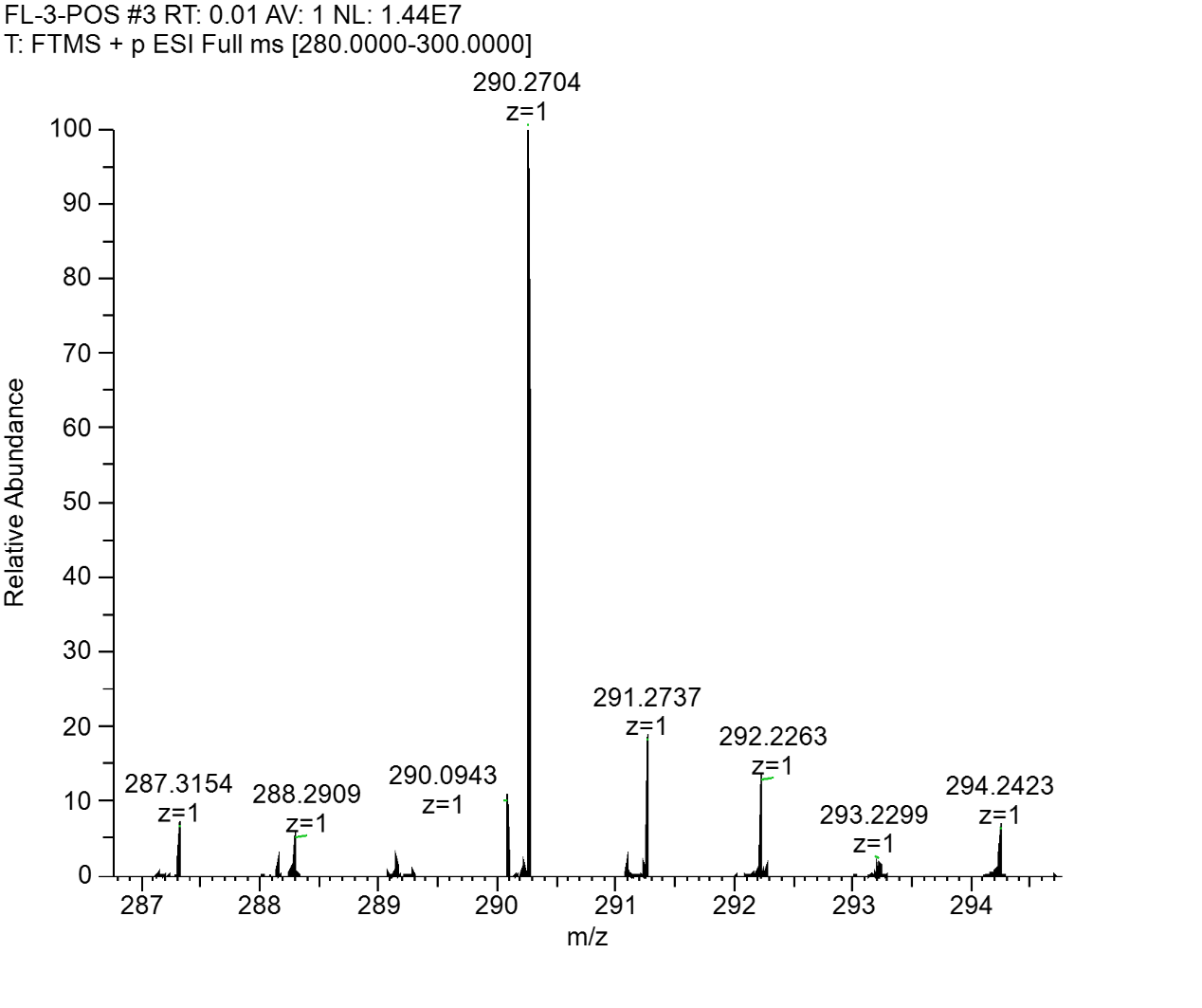


Figure S11. The (A) nHA, (B) nHD, (C) LogP, (D) Molecular weight and (E) TPSA of molecules in the three datasets.


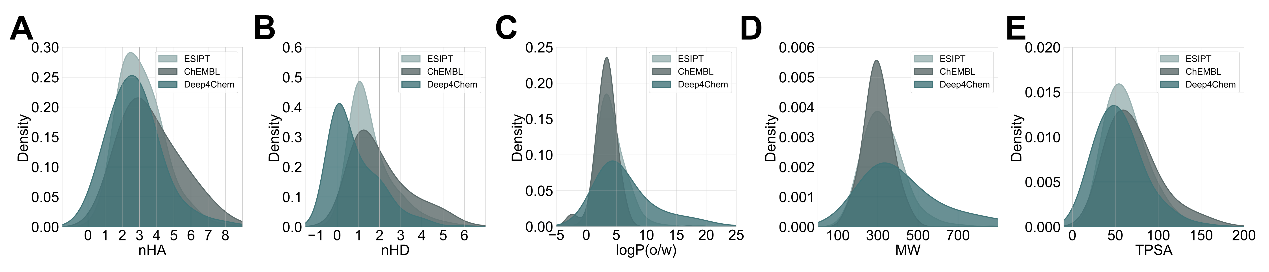


Figure S12. Feature selection based on RFE CV-RF for the E-CM model, with cross validation score (accuracy) as the evaluation metric.


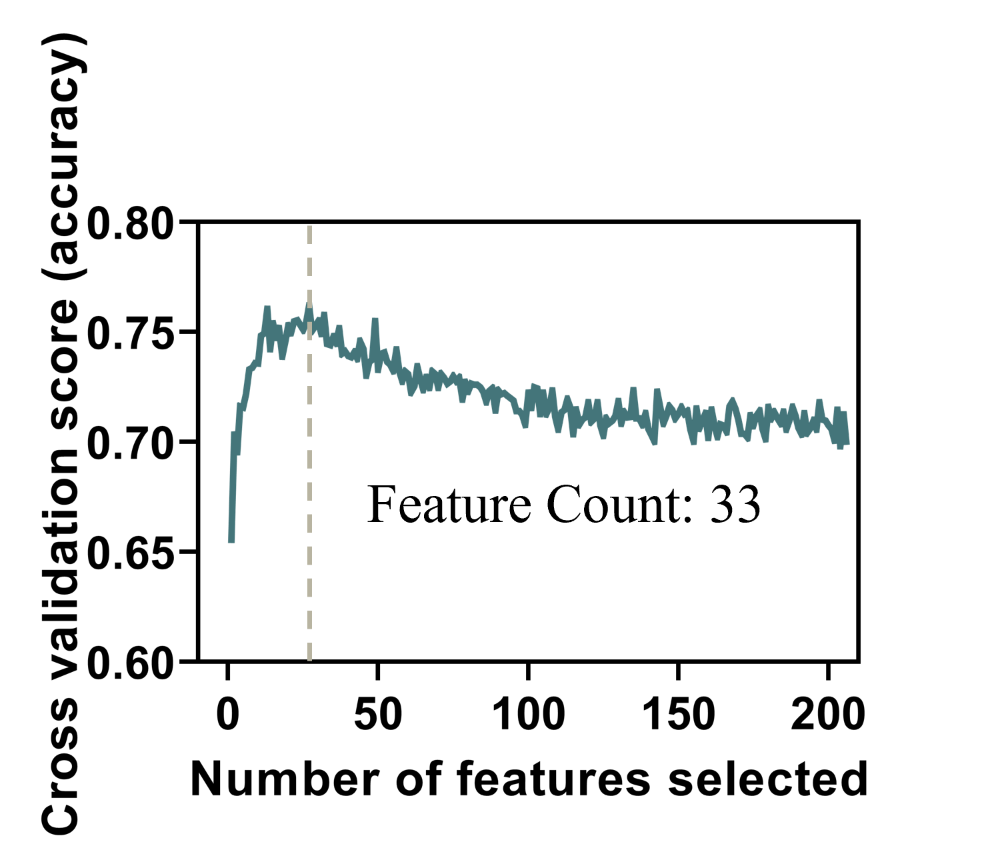


Figure S13. Feature selection based on RFE CV-RF for the E-FL model, with cross validation score (accuracy) as the evaluation metric.


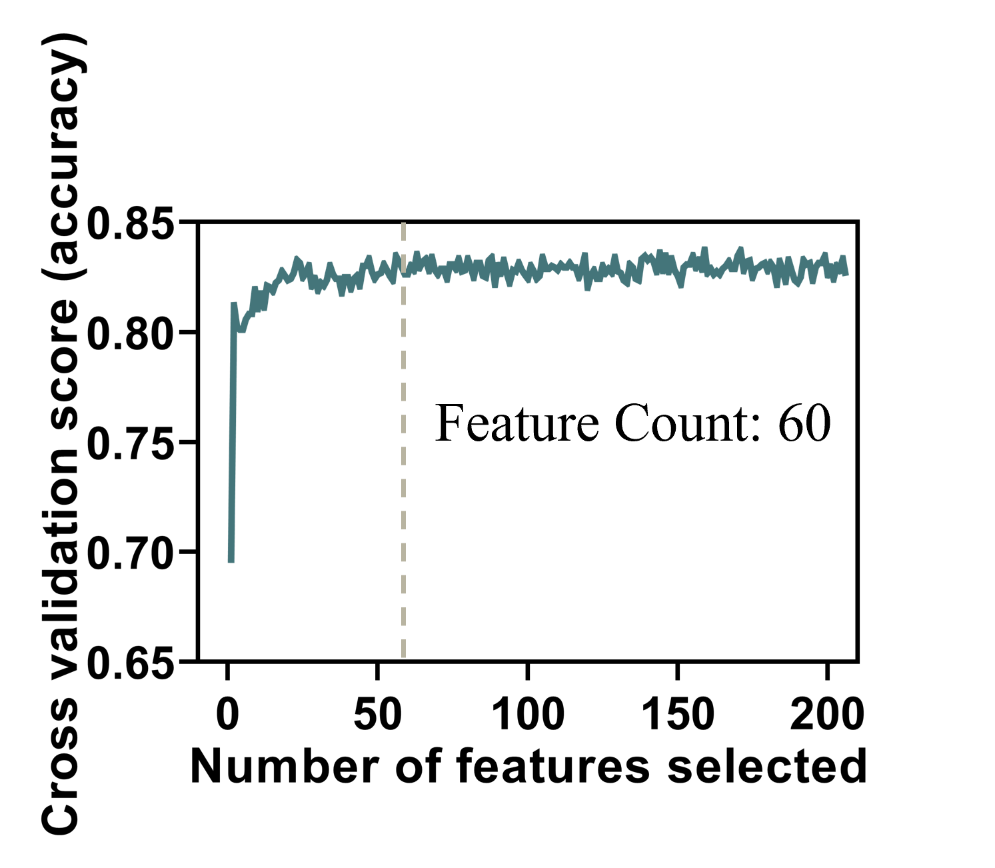


**Figure S14. Feature selection based on RFE CV-RF for the E-Barrier model, with R^2^ as the evaluation metric.**


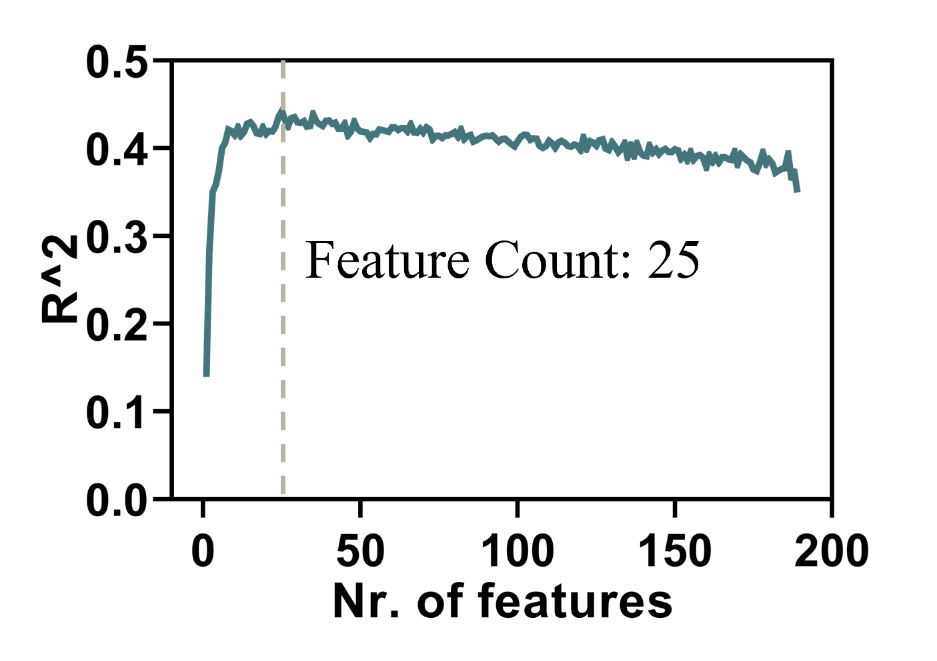

Supplement: Supplementary file 1 — Supporting Information [file ADVS-11-2405596-s001.docx]
